# Epistatic Interactions in Genetic Regulation of t-PA and PAI-1 Levels in a Ghanaian Population

**DOI:** 10.1371/journal.pone.0016639

**Published:** 2011-01-31

**Authors:** Nadia M. Penrod, Kwabena A. Poku, Douglas E. Vaughn, Folkert W. Asselbergs, Nancy J. Brown, Jason H. Moore, Scott M. Williams

**Affiliations:** 1 Department of Genetics, Dartmouth Medical School, Lebanon, New Hampshire, United States of America; 2 Human Services Management and Public Administration, Business School, University of Ghana, Legon, Ghana; 3 Division of Cardiovascular Medicine, Department of Medicine, Vanderbilt University Medical School, Nashville, Tennessee, United States of America; 4 Division Heart and Lungs, Department of Cardiology, University Medical Center Utrecht, Utrecht, The Netherlands; 5 Division of Clinical Pharmacology, Department of Medicine, Vanderbilt University Medical School, Nashville, Tennessee, United States of America; 6 Department of Community and Family Medicine, Dartmouth Medical School, Lebanon, New Hampshire, United States of America; 7 Department of Molecular Physiology and Biophysics, Center for Human Genetics Research, Vanderbilt University, Nashville, Tennessee, United States of America; University of Miami, United States of America

## Abstract

The proteins, tissue plasminogen activator (t-PA) and plasminogen activator inhibitor 1 (PAI-1), act in concert to balance thrombus formation and degradation, thereby modulating the development of arterial thrombosis and excessive bleeding. PAI-1 is upregulated by the renin-angiotensin system (RAS), specifically by angiotensin II, the product of angiotensin converting enzyme (ACE) cleavage of angiotensin I, which is produced by the cleavage of angiotensinogen (AGT) by renin (REN). ACE indirectly stimulates the release of t-PA which, in turn, activates the corresponding fibrinolytic system. Single polymorphisms in these pathways have been shown to significantly impact plasma levels of t-PA and PAI-1 differently in Ghanaian males and females. Here we explore the involvement of epistatic interactions between the same polymorphisms in central genes of the RAS and fibrinolytic systems on plasma t-PA and PAI-1 levels within the same population (n = 992). Statistical modeling of pairwise interactions was done using two-way ANOVA between polymorphisms in the ETNK2, RENIN, ACE, PAI-1, t-PA, and AGT genes. The most significant interactions that associated with t-PA levels were between the ETNK2 A6135G and the REN T9435C polymorphisms in females (p = 0.006) and the REN T9435C and the TPA I/D polymorphisms (p = 0.005) in males. The most significant interactions for PAI-1 levels were with REN T9435C and the TPA I/D polymorphisms (p = 0.001) in females, and the association of REN G6567T with the TPA I/D polymorphisms (p = 0.032) in males. Our results provide evidence for multiple genetic effects that may not be detected using single SNP analysis. Because t-PA and PAI-1 have been implicated in cardiovascular disease these results support the idea that the genetic architecture of cardiovascular disease is complex. Therefore, it is necessary to consider the relationship between interacting polymorphisms of pathway specific genes that predict t-PA and PAI-1 levels.

## Introduction

Preventing excessive blood loss following vessel injury is controlled through a process known as hemostasis. The tight regulatory mechanisms of hemostasis manage this response to prevent thrombus formation. One mechanism involved in this balance is fibrinolysis, the physiological breakdown of fibrin, an essential component of blood clots. Upon release of the serine protease tissue plasminogen activator (t-PA) from vascular endothelial cells, circulating plasminogen is cleaved to produce plasmin, a proteolytic enzyme responsible for the degradation of fibrin [1]. The fibrinolytic pathway is also partially regulated by the renin-angiotensin system; this has been demonstrated by studies that show pharmacological inhibition of the angiotensin converting enzyme (ACE) indirectly stimulates release of t-PA [2] while reducing levels of its inhibitor, plasminogen activator inhibitor-1 (PAI-1) [3]. Other studies have found comparable effects of the renin-angiotensin system (RAS) on both t-PA and PAI-1, a connection that may be modulated by genetic variation in both systems [2], [4]–[6]. Clinical evidence indicates that plasma concentrations of t-PA and PAI-1 can also serve as biomarkers of first myocardial infarction [7], [8], atherosclerosis [9], ischemic stroke [10] and ischemic heart disease [11].

In the present study, polymorphisms have been selected from genes in the renin-angiotensin and fibrinolytic pathways to assess the genetic contribution to the protein concentrations of t-PA and PAI-1 in a large-scale population-based cohort; this study supplements the available literature that is primarily based on high-risk populations. To this end, we have designed complementary population based studies in the Netherlands and Ghana [12]–[17]. In prior analysis of the Ghanaian sample inter-individual variation in plasma levels of t-PA and PAI-1 was associated with conventional cardiovascular risk factors including body mass index (BMI), blood pressure, cholesterol, glucose and triglycerides [16], [17]. Additionally, genetic analyses revealed statistically significant associations between polymorphisms in the RAS genes as well as t-PA and PAI-1 on plasma concentrations of the t-PA and PAI-1 proteins. This univariate analysis demonstrated that t-PA and PAI-1 concentrations are influenced by both traditional cardiovascular risk factors as well as genetic polymorphisms and that these factors differ between females and males [16]. The current study was conducted to explore the role of two-way epistatic interaction effects on the plasma levels of the t-PA and PAI-1 proteins based on polymorphisms in genes from the renin-angiotensin and fibrinolytic pathways in a large-scale population-based sample from urban West Africa, and to assess whether such analyses are significantly more informative than single SNP analyses. The hypothesis of interaction among these genes/markers is not only supported by previous statistical work [13], but by data on protein-protein interactions [18].

## Materials and Methods

### Study Population

The population-based sample from urban West Africa analyzed in this study has been described previously [17]. In summary, participants were enrolled by word-of-mouth in Sunyani, Ghana, the capital city of the Brong-Ahafo region (n = 992) regardless of chronic disease status. Exclusion criteria comprised age less than 18 years, prior enrollment of a first or second degree relative, and indication of acute illness that could impact t-PA or PAI-1 levels. Women represented 57.3% of the sample. Protocols and consent forms were approved by both U.S. and Ghanaian authorities in accordance with the Declaration of Helsinki.

### Laboratory Measurements

Blood samples were collected for measurement of fasting glucose, lipids, t-PA and PAI-1 levels by commercially available assays, protocols have been described elsewhere [17]. The t-PA and PAI- 1 measures will be discussed here in brief. To address circadian rhythm derived variability in t-PA and PAI-1 protein concentrations, all blood samples were collected between 8:00 and 10:00 am. Day-to-day variability in these proteins was not addressed in the current study. Blood was collected in Biopool Stabilyte tubes (Umea, Sweden) and chilled on ice briefly before centrifugation at 3000 Gs and 0°C for 20 minutes. Plasma was then transferred under sterile conditions into cryotubes and immediately stored in liquid nitrogen for shipment to Vanderbilt University (Nashville, TN) in IATA approved Cryo shippers (MVE products). Plasma t-PA and PAI- 1 levels were measured, using a commercially available enzyme-linked immunoassay (ELISA, Biopool AB, Umea, Sweden).

### Measurement of Genetic Polymorphisms

DNA isolation was carried out on-site using PureGene DNA purification kits (Gentra Systems). Polymorphisms were detected using TaqMan probes and PCR primers designed through the Assays-by-Design service of Applied Biosystems. TaqMan SNP Genotyping Assays were processed and analyzed according to the manufacturer's recommendations with an ABI 7900 HT Fast Real Time PCR system and the SDS 2.0 genotype calling software (Applied Biosystems), respectively. The nine polymorphisms genotyped are listed in Table 1. Due to a rare genotype, rs5186 (MAF  = 0.02), was excluded from the analysis. The eight remaining polymorphisms were used for the interaction analyses.

**Table 1:** Genotyped polymorphisms.

Gene	rs	Chromosome	Position^1^	MinorAllele	Minor Allele Frequency
Angiotensin converting enzyme (ACE I/D)	rs4646994	17	61565904	I	0.43
Angiotensin II receptor, type 1	rs5186	3	148459988	C	0.02
Angiotensinogen (AGT M235T)	rs699	1	230845794	A	0.08
Ethanolamine kinase 2 (ETNK2 C5047T)	rs2293337	1	204115934	T	0.15
Ethanolamine kinase 2 (ETNK2 A6135)	rs1917542	1	204114846	G	0.40
Renin (REN T9435C)	rs3730103	1	204125987	C	0.33
Renin (REN G6567T)	rs1464816	1	204128854	T	0.36
t-PA (TPA I/D)	rs4646972	8	42039905	I	0.36
PAI-1 (PAI 4G/5G)	rs1799768	7	100769706	4G	0.20

The angiotensin II receptor, type 1 polymorphism was excluded from interaction analyses due to low minor allele frequency of the rare allele.

1 Build 37.1.

The variants selected for this study included two polymorphisms in the renin gene (REN), two polymorphisms in the ethanolamine kinase gene (ETNK2), and one in each of the angiotensinogen (AGT), ACE, t-PA and PAI-1 genes. Note that although ETNK2 is not expected to affect fibrinolytic balance per se, the SNPs in this gene were chosen because ETNK2 is less than 5 KB from REN, and these may represent or tag variants in REN or variants that affect REN expression. All of these genes have been associated with cardiovascular endpoints or as functional variants associated with plasma PAI-1 and t-PA concentrations [16]. Also, these SNPs were analyzed because they are the same ones previously reported in our single marker analysis and their analyses allowed direct comparison of the single SNP to two SNP analyses [16].

### Statistical Analysis

Baseline characteristics for continuous and categorical data have been reported as mean ± standard deviation and as group percentages, respectively. Comparisons of continuous characteristics between males and females were done using the Student's t test. Comparisons of categorical characteristics between males and females were done with the χ^2^ test. The baseline characteristics of the population-based sample are provided in Table 2. It has been demonstrated previously that anthropometric, metabolic and genetic factors show statistically significant variation between the sexes, as such, further analyses have been carried out in males and females separately [16].

**Table 2:** Baseline characteristics.

Characteristic	Females	Males	p-value‡
	(n = 569)	(n = 423)	
Age (years)	42.7±10.9	44.0±12.7	0.074
Height (cm)	162±6.33	172±7.04	<0.001
Weight (kg)	70.2±14.7	70.7±12.9	0.550
BMI(kg/m2)	26.6±5.34	23.8±4.15	<0.001
CurrentSmoking	0.00%	3.10%	<0.001
SBP(mmHg)	126±18.9	132±20.8	<0.001
DBP(mmHg)	77.3±10.5	78.0±13.0	0.380
Glucose(mg/dl)	94.5±24.7	91.5±21.1	0.051
Totalcholesterol(mg/dl)	182±42.7	170±45.4	<0.001
Triglycerides(mg/dl)*	4.34±0.509	4.42±0.542	0.006
t-PA(ng/ml)*±	1.80±0.667	1.78±0.805	0.690
PAI-1(ng/ml)*	1.27±1.33	1.03±1.49	0.008

Participants were enrolled by word-of-mouth in Sunyani, Ghana, the capital city of the Brong-Ahafo region (n = 992).

*indicates ln transformed value.

The purpose of the statistical analysis in the current study was to assess the role combinations of genetic polymorphisms, within the renin-angiotensin and fibrinolytic pathways, play in the prediction of plasma t-PA and PAI-1 levels. The null hypothesis being tested is that the effect of any single polymorphism on t-PA and PAI-1 plasma levels is independent of the effects of any other polymorphism. To test the null hypothesis we used two-way analysis of variance (ANOVA) to calculate an interaction term. The dependent variable (t-PA or PAI-1) has been natural log transformed for all analyses for normalization as previously reported [16]. The results were considered statistically significant if the p-value was less than or equal to 0.05 and marginally significant at the 0.10 level. Both thresholds were considered evidence toward rejection of the null hypothesis in favor of the alternative hypothesis of epistasis or gene-gene interaction effects. In addition, we calculated r^2^ values of the interaction term in each statistically significant model as an estimate of the amount of variability explained by the SNP pairs. All tests were carried out in males and females separately as the distribution of PAI-1 have been previously shown to be sex specific [12], [16].

More specifically, all genotypes were coded to allow for assessment of additive effects (AA  = 0, Aa  = 1, aa  = 2), dominant effects (AA  = 0, Aa  = 0, aa  = 1) and recessive effects (AA  = 0, Aa  = 1, aa  = 1). Using ANOVA, all 28 possible pairs of genotypes (all combinations of 8 choose 2) were evaluated for nine possible interaction effects including additive by additive, additive by dominant, additive by recessive, dominant by additive, dominant by dominant, dominant by recessive, recessive by additive, recessive by dominant and recessive by recessive. In total, 970 tests were performed. After adjusting for missing genotypes following the conversion of the genotype coding to account for the various interaction effect models, 243 tests were carried out in females per phenotype and 242 tests were carried out in males per phenotype. We set three thresholds of significance for making inferences about the epistatic effects of the polymorphisms. First, we use an uncorrected significance level of α = 0.10 to indicate suggestive evidence for an interaction. This liberal threshold was set as an exploratory cut-off with consideration for the known role that the pathways and genes included in this study play in t-PA and PAI-1 biochemistry and the likelihood that the chosen polymorphisms are strong candidates. Second, we use an uncorrected significance level of α = 0.05 to indicate evidence for an interaction. Our most stringent condition is a significance level of α = 0.025. This significance threshold represents a correction for the different genetic models tested and was derived from 1000 Monte Carlo simulations under the null hypothesis and has been described elsewhere [13]. Results for all tests are reported in the supplementary material so that interpretation of statistical significance as it relates to biological significance can be assessed by the reader. Corrections were not made for multiple testing among variants since there is clear biological evidence that each pathway represented by the candidate SNPs in this study is involved in the regulation of t-PA and PAI-1 expression either directly or indirectly meaning that the universal null hypothesis of association by random chance does not apply [19]. Additionally, since ANOVA has more power to detect independent main effects than interactions effects for this exploratory analysis we are more concerned with type II errors (false-negatives) than type I errors (false-positives).

As the final step in this study we selected a subset of the results containing the 112 most significant interaction pairs (i.e. 28 possible genotype pairs by sex and phenotype) to create a new data set of minimum p-values from the set of different genotype codings. Because we selected for rare or extreme events we would not expect these data to follow a normal distribution. As such, this subset of the results was modeled by fitting the generalized extreme value distribution. The parameters of this distribution are location, scale, and shape. Using the maximum likelihood estimates of these parameters from the model we calculated the probabilities of getting the observed p-values for the most significant interacting pairs of SNPs. This analysis was carried out in R using the package extremes (http://cran.rproject.org/web/packages/extRemes/index.html).

## Results

**Figure 1:**
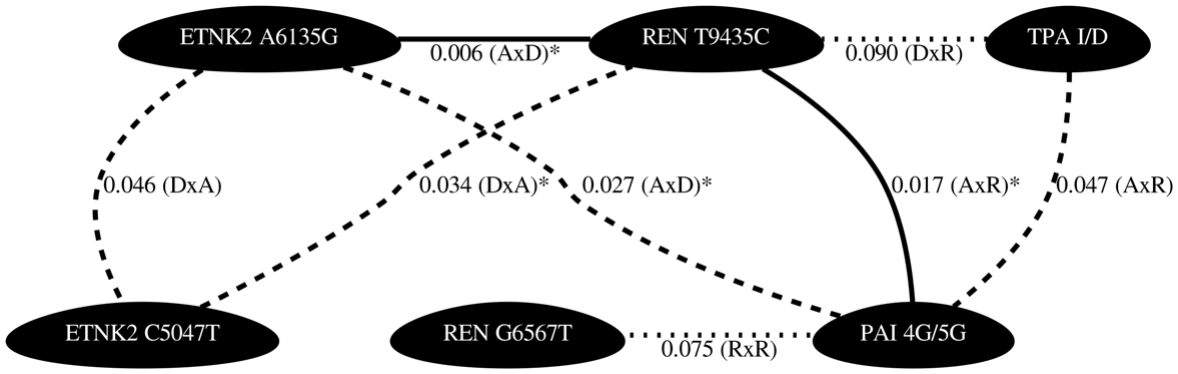
Network diagrams to summarize the results of the statistical analysis of epistasis between polymorphisms within the renin-angiotensin and fibrinolytic systems on the levels of t-PA in the plasma of females. Three levels of statistical significance are represented in these figures, suggestive evidence where p<0.10, denoted by dotted lines, evidence where p<0.05, denoted by dashed lines and strong evidence where p<0.025, denoted by solid lines. The * symbol indicates results that have been validated by extreme value analysis. When the same pairs were statistically significant under various genotype codes (e.g. additive by additive vs. dominant by additive) only the most significant result was chosen for display in the network diagrams to visually simplify the plots. Female REN G6567T genotypes have previously been shown to associate with t-PA in single gene analyses.

**Figure 2:**
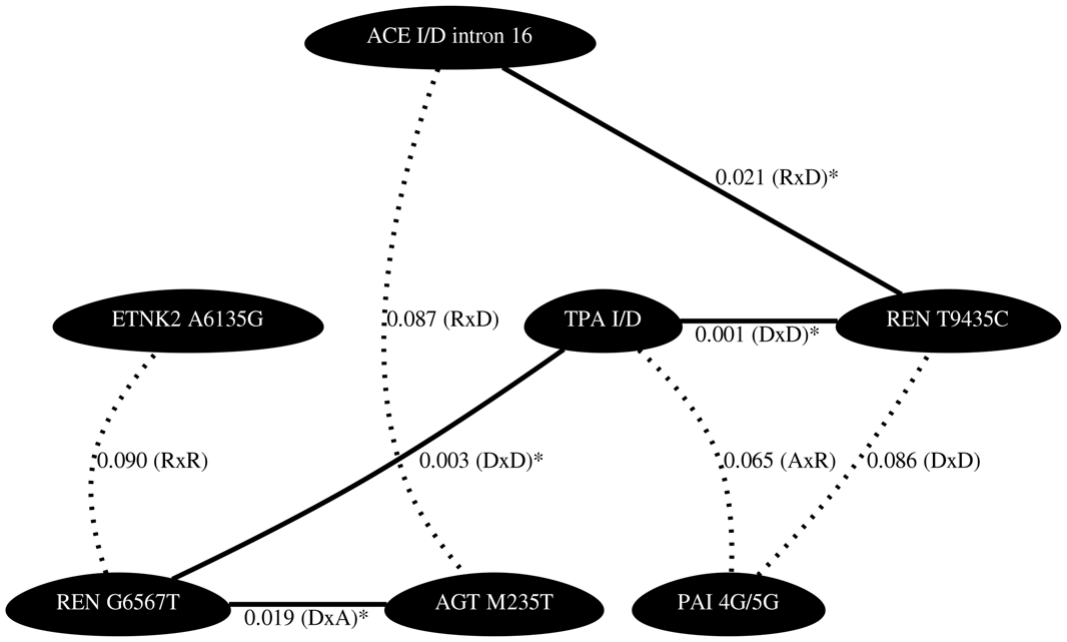
Network diagrams representing polymorphism interactions predictive of plasma t-PA in males. Figure is as described for figure 1. TPA I/D and REN G6567T genotypes in males have previously been shown to associate with t-PA in single gene analyses.

**Figure 3:**
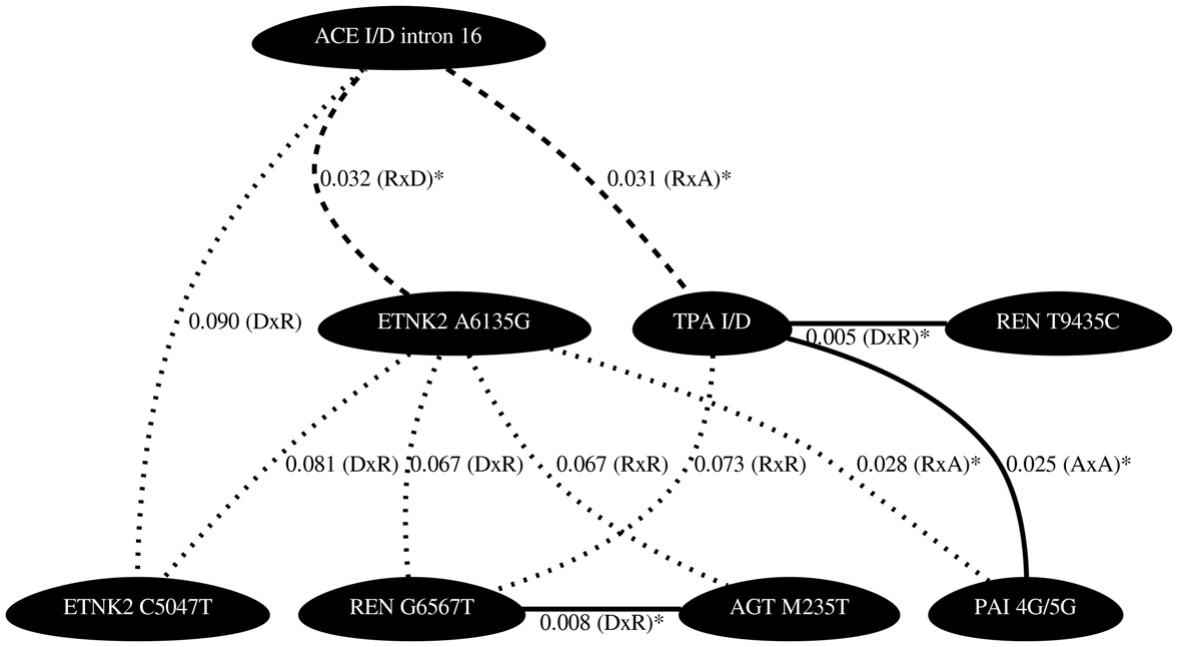
Network diagrams representing polymorphism interactions predictive of plasma PAI-1 in females. Figure is as described for figure 1. Female PAI 4G/5G genotypes have previously been shown to associate with PAI-1 in single gene analysis.

**Figure 4:**
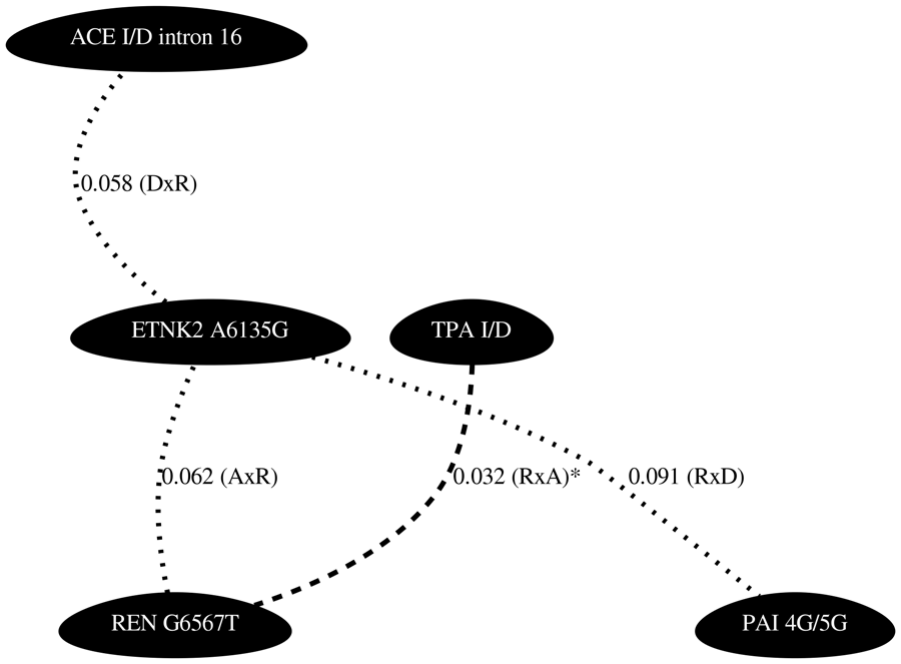
Network diagrams representing polymorphism interactions predictive of plasma PAI-1 in males. Figure is as described for figure 1. Male ETNK2 A6135G, TPA I/D, and REN G6567T genotypes have previously been shown to associate with PAI-1 in single gene analysis.

A summary of the results are presented as network diagrams in Figures 1, 2, 3, 4. The most significant findings are illustrated without redundancy, meaning that if the same two SNPs in combination met our threshold criteria under different models (e.g. DxA and DxR) that SNP combination was only represented one time.

In females, we found significant interaction effects between polymorphisms in various components of the renin-angiotensin pathways predictive of plasma t-PA levels. Of the 28 pairwise combinations tested, 8 interactions met our significance criteria (Figure 1 and 1). The most significant of these gene-gene interactions occurred between the ETNK2 A6135G variant and the REN T9435C variant (p = 0.006, r^2^ = 1.82%), but the largest r^2^ was for an interaction between markers REN T9435C and ETNK2 A6135G (p = 0.011, r^2^ = 2.27%) (Table S2). We also found significant interaction effects between polymorphisms in various components of the renin-angiotensin pathways predictive of plasma PAI-1 levels. Of the 28 pairwise combinations tested, 8 interactions met our significance criteria (Figure 2 and Table S3). The most significant of these gene-gene interactions occurred between the REN T9435C variant and the TPA I/D variant (p = 0.001, r^2^ = 1.98%)(Table S2). Interactions occurring between REN T9435C and TPA I/D, REN T9435C and PAI 4G/5G, as well as TPA I/D and PAI 4G/5G were significant for both t-PA and PAI-1 levels in females (Figures 1 and 2). It is of note that for all models presented the r^2^ for the interaction term accounted for the majority of the variance explained in the full model (Table S2).

In males, we found significant interaction effects between polymorphisms in various components of the renin-angiotensin pathways predictive of plasma t-PA levels. Of the 28 pairwise combinations tested, 11 interactions met our significance criteria (Figure 3 and Table S4). The most significant of these gene-gene interactions occurred between the TPA I/D variant and the REN T9435C variant (p = 0.005, r^2^ = 1.80%) (Table S2). The largest r^2^ for t-PA was for an interaction between markers TPA ID and PAI-1 4G5G (p = 0.025, r^2^ = 2.62%)(Table S2). We also found significant interaction effects between polymorphisms in various components of the renin-angiotensin pathways predictive of plasma PAI-1 levels. Of the 28 pairwise combinations tested, 4 met our significance criteria (Figure 4 and Table S5). The most significant of these gene-gene interactions occurred between the TPA I/D variant and the REN G6567T variant (p = 0.032, r^2^ = 1.08%) (Table S2). Interactions occurring between ACE I/D intron 16 and ETNK2 A6135G, ETNK2 A6135G and REN G6567T, ETNK2 A6135G and PAI 4G/5G, as well as TPA I/D and REN G6567T were associated with both t-PA and PAI-1 levels in males.

Focusing on the results displayed in the network plots (Figures 1, 2, 3, 4) we can compare the interactions found to predict t-PA and PAI-1 levels across the sexes. The female and male data revealed the following common interacting partners in the analyses for t-PA: ETNK2 A6135G and ETNK2 C5047T, ETNK2 A6135G and PAI 4G/5G, REN T9435C and TPA I/D, in addition to TPA I/D and PAI 4G/5G. Interaction pairs common to females and males in the PAI-1 analyses included: ETNK2 A6135G interacting with REN G6567T and TPA I/D interacting with REN G6567T.

While some of the interactions are shared between the sexes, there are also many differences. For example, we have strong evidence supporting an interaction effect of ETNK2 A6135G and REN T9435C on plasma t-PA levels in females (p = 0.006) while there is no supporting evidence in males (p = 0.613). Similarly, we see strong evidence for an interaction effect of REN G6567T and AGT M235T on plasma t-PA levels in males (p = 0.008) but not in females (p = 0.496). The same is true when considering the strong evidence in support of an interaction effect between TPA I/D and REN T9435C on plasma levels of PAI-1 in females (p = 0.001) compared to that in males (p = 0.971).

**Figure 5:**
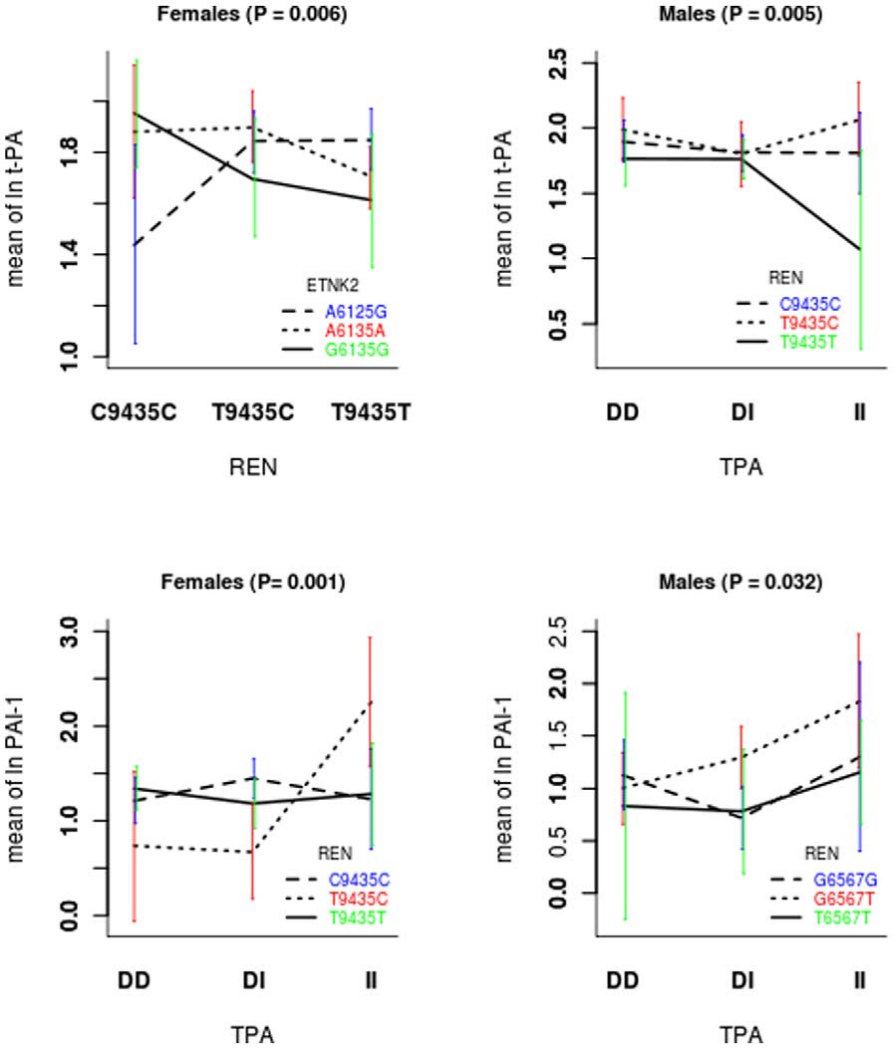
Interaction plots showing the most significant interacting polymorphisms in association with the natural log-transformed values. A) t-PA in males with t-PA I/D and REN C9435T genotypes; B) t-PA in females with ETNK2 A6135G and REN C9435T; C) PAI-1 in males with REN G6567T and t-PA I/D; D) PAI-1 in females with REN C9435T and t-PA I/D. These plots reflect the mean log transformed protein levels observed for the individual polymorphism combinations where deviation from parallel between the lines indicates dependency. Actual values are given in Table S6.

It is also of interest to examine the structure of these interactions. Interaction plots of the most significant findings for each sex and phenotype pair are presented in Figure 5. Additional plot information including sample size and confidence intervals is presented in Table S6. The deviations from parallel in these plots indicate that, in all cases, the protein levels of both t-PA and PAI-1 depend on genotype combinations and not single SNP status, in other words, epistatic interactions.

Here we have described and illustrated the 31 most significant gene-gene interactions. However, a total of 86 of the 970 tests were significant or marginally significant (p<0.10). Of the most significant models three were characterized as dominant-dominant interactions, five were characterized as dominant-additive interactions, eleven were characterized as dominant-recessive interactions, one was characterized as an additive-additive interaction, seven were characterized as recessive-additive interactions, and the remaining four were characterized as recessive-recessive interactions.

## Discussion

The fibrinolytic system maintains balance through a series of enzymatic reactions, regulating the dissolution of thrombi. The principal regulators of the system are t-PA and its primary inhibitor PAI-1. Furthermore, crosstalk exists between the fibrinolytic pathway and the renin-angiotensin pathway providing an additional layer of regulation. In this study we investigated the effects of epistatic interactions, between polymorphisms within genes of t-PA, PAI-1 and the renin-angiotensin system, on the plasma concentration of the t-PA and PAI-1 proteins. Our results support the idea that the genetic architecture underlying cardiovascular disease is complex and requires consideration of multiple interacting polymorphisms in concert to reliably predict the levels of associated proteins, t-PA and PAI-1.

In a previously published study we reported that anthropometric, metabolic, and genetic cardiac risk factors, in this population, are sex-dependent [16]. Although we did not consider covariates in the present study, we chose to carry-out the analyses separately for females and males because one of the dependent variables, PAI-1 levels, was one of the factors that differed between the sexes. We also wanted to maintain consistency between this analysis and the prior studies based not only on this population, but also those based on a cohort from the Netherlands. As expected, we found supporting evidence for male-female differences in the underlying genetic factors predictive of t-PA and PAI-1 plasma concentrations. In fact, the majority of interactions were sex specific. This result is consistent with the results of an epistasis analysis done with population data from the Netherlands where similar genetic differences were found to be predictive of t-PA and PAI-1 levels for each sex [13].

Evaluation of all pairwise combinations of the eight measured polymorphisms revealed many statistical interactions involving polymorphisms within the renin-angiotensin system, as well as interactions involving polymorphisms between the renin-angiotensin system and polymorphisms in the t-PA or PAI-1 genes. Because this was an exploratory analysis we tested the 28 possible pairwise interactions per sex and phenotype combination across each of the 9 interaction models but selected only the single best model per interacting pair. We chose not to correct for multiple testing but instead to validate our findings by using extreme value analysis. We found that 15 of the 31 observed p-values representing pairwise interactions had a probability of less than 10% while 5 of the observed p-values had a probability of less than 5%. Based on these results we believe that at a minimum these 15 pairwise interactions represent true statistical epistasis. These validated interactions coincide with the most statistically significant results illustrated in Figures 1, 2, 3, 4. It is of note that when compared to the single SNP analyses where only 5 of the 8 variants were shown to individually associate with t-PA and/or PAI-1 concentrations, even with liberal statistical criteria [16], we found that all 8 variants were significant in the 2-locus analyses. For example, REN T9435C and ETNK2 A6135G were predictive of female t-PA levels together but neither SNP alone showed association. Based on these results it is reasonable to conclude that these interactions, involving polymorphisms lacking an independent main effect, will be necessary to inform complete genotype to phenotype mapping.

In addition to reporting p-values we have also reported r^2^ values for all statistically significant interacting partners. These values serve as an estimate of the amount of variability explained by each interaction in each model. Our two-locus interaction terms explain as much as 2.6% of the variability in protein levels, but even these modest effect sizes are larger than those of the single SNP analyses (less than 1.3%, [16]. Interestingly, we often see the highest r^2^ values for interactions among pairs of SNPs that were not individually associated with either plasma t-PA or PAI-1. For example, the interaction between REN T9435C and ETNK2 A6135G in females predicts t-PA levels with a p-value of 0.010 and a corresponding r^2^ value of 2.27%, but neither SNP alone showed an association with female t-PA levels. One exception to this observation is the evaluation of male t-PA levels where TPA I/D (p = 0.026, r^2^ = 1.20%) and REN T9435C (p = 0.019, r^2^ = 1.30%) do show individual associations, but, notably, when combined in a two-way model the correlation becomes stronger (p = 0.002, r^2^ = 4.45% for the full model and p = 0.016, r^2^ = 1.92% for the interaction alone). If these two SNPs had independent main effects on t-PA levels we would expect the r^2^ value of the two-way model to equal the sum of the individual r^2^ values. It is important to note that the complete genetic model will likely include main effects, numerous gene-gene and gene-environment interactions Nonetheless, in some cases we are able to explain more than 2% of the variability in protein levels just by focusing on two-locus interaction terms. This underscores the necessity of considering gene-gene interactions when studying common human phenotypes/diseases.

The variants ETNK2 A6135G, REN G6567T, TPA I/D, and PAI 4G/5G, were the most frequently observed in our models, appearing in all of the network diagrams. Of these variants ETNK2 A6135G is involved in the most statistically significant interactions. The ETNK2 gene codes for a protein that catalyzes the first reaction the CDP-ethanolamine pathway, resulting in production of the major membrane lipid phosphatidylethanolamine (PtdEtn), this gene was chosen not for its function but because it is located on chromosome 1q32 near the renin gene and may include alternative promoter regions for renin. It is known that ETNK2 −/− mice often have failed fetal development at the late stages of pregnancy due to extensive placental thrombosis, suggesting a critical role for ETNK2 sequences in hemostasis [20]. Another commonly appearing variant also supported in the literature, in association with PAI-1 levels, is the PAI 4G/5G variant. This polymorphisms shows a functional role in basal transcription levels of PAI-1 associating with increased PAI-1 plasma levels [21], [22].

By evaluating all pair wise combinations of these polymorphisms for association with t-PA and PAI-1 protein levels we have begun to assess the contribution statistical epistasis plays in defining genetic risk factors of cardiovascular disease. And because these genes have good prior evidence for biological interaction [2], [3], the results, even when considering multiple testing, provide a plausible scenario for the regulation of PAI-1 and tPA. However, we recognize that these results do not permit us to draw conclusions regarding biological function or causation because statistical epistasis at the population level and biological epistasis at the individual/biochemical levels are not identical [23]. Nevertheless, we have shown that, at the population level, pairs of polymorphisms consistently provide more information on t-PA and PAI-1 protein levels than any single polymorphism, and these pairwise combinations provide testable hypotheses that can be addressed experimentally. Taken together these results support the hypothesis that genetic architecture of cardiovascular risk is complex, but can be elucidated.

## Supporting Information

Table S1 ***p*** **-values for epistatic effects between polymorphisms in association with plasma t-PA levels for females.** All nine interaction models are presented. Each table displays the results of one interaction pairing as indicated in the upper left-hand corner. The notation is column by row, for example, a DxA interaction indicates that the SNPs across the columns are encoded as dominant while the SNPs down the rows are endoded as additive. *p*-values <0.10 are displayed in boldface. D  =  dominant, A  =  additive, R  =  recessive.
(DOC)

Table S2 A. Variability (r^2^) of t-PA and PAI-1 levels explained by models combining two SNPs without an interaction term (SNP1 + SNP2), models combining two SNPs with an interaction term (SNP1*SNP2) and the variability obtained by extracting only the interaction term from the full model in females. Results shown meet our exploratory statistical threshold of p < 0.10 for the interaction term from the full model. * denote single SNP analysis (see Schoenhard et al. 2008 [16]). D  =  dominant encoding, A  =  additive encoding, R  =  recessive encoding.
(DOC)

Table S3 ***p*** **-values for epistatic effects between polymorphisms in association with plasma PAI-1 levels for females.** All nine interaction models are presented. Each table displays the results of one interaction pairing as indicated in the upper left-hand corner. The notation is column by row, for example, a DxA interaction indicates that the SNPs across the columns are encoded as dominant while the SNPs down the rows are endoded as additive. *p*-values <0.10 are displayed in boldface. D  =  dominant, A  =  additive, R  =  recessive.
(DOC)

Table S4 ***p*** **-values for epistatic effects between polymorphisms in association with plasma t-PA levels for males.** All nine interaction models are presented. Each table displays the results of one interaction pairing as indicated in the upper left-hand corner. The notation is column by row, for example, a DxA interaction indicates that the SNPs across the columns are encoded as dominant while the SNPs down the rows are endoded as additive. *p*-values <0.10 are displayed in boldface. D  =  dominant, A  =  additive, R  =  recessive.
(DOC)

Table S5 ***p*** **-values for epistatic effects between polymorphisms in association with plasma PAI-1 levels for males.** All nine interaction models are presented. Each table displays the results of one interaction pairing as indicated in the upper left-hand corner. The notation is column by row, for example, a DxA interaction indicates that the SNPs across the columns are encoded as dominant while the SNPs down the rows are endoded as additive. p-values <0.10 are displayed in boldface. D  =  dominant, A  =  additive, R  =  recessive.
(DOC)

Table S6 **Description of the data underlying the points in the interaction plots displayed in** **Figure 5** **.** The mean protein level, 95% confidence interval and number of individuals for each genotype combination (i.e. each data point of interaction plot) is presented.
(DOC)
