# Correction: Epistatic Interactions in Genetic Regulation of t-PA and PAI-1 Levels in a Ghanaian Population

**DOI:** 10.1371/annotation/2501cac9-f313-406f-919b-314f12b43516

**Published:** 2011-02-11

**Authors:** Nadia M. Penrod, Kwabena A. Poku, Douglas E. Vaughn, Folkert W. Asselbergs, Nancy J. Brown, Jason H. Moore, Scott M. Williams

The third author's name is misspelled. The correct spelling is: Douglas E. Vaughan. The correct citation is: Penrod NM, Poku KA, Vaughan DE, Asselbergs FW, Brown NJ, et al. (2011) Epistatic Interactions in Genetic Regulation of t-PA and PAI-1 Levels in a Ghanaian Population. PLoS ONE 6(1): e16639. doi:10.1371/journal.pone.0016639

