# Supplementary material for: Epistatic Interactions in Genetic Regulation of t-PA and PAI-1 Levels in a Ghanaian Population
Source: PLoS One. 2011 Jan 31;6(1):e16639. doi: 10.1371/journal.pone.0016639 (PMC3031598; doi:10.1371/journal.pone.0016639)
Supplement: Table S1 — p -values for epistatic effects between polymorphisms in association with plasma t-PA levels for females. All nine interaction models are presented. Each table displays the results of one interaction pairing as indicated in the upper left-hand corner. The notation is column by row, for example, a DxA interaction indicates that the SNPs across the columns are encoded as dominant while the SNPs down the rows are endoded as additive. p-values <0.10 are displayed in boldface. D = dominant, A = additive, R = recessive. (DOC) [file pone.0016639.s001.doc]

**Table S1**.

| **DxA** | *ACE ID* | AGT  *M235T* | *PAI-1 4G5G* | REN G/T  *rs1464816* | ETNK2 A/G  *rs1917542* | ETNK2 C/T  *rs2293337* | REN T/C  *rs3730103* | *t-PA ID* |
| --- | --- | --- | --- | --- | --- | --- | --- | --- |
| *ACE_ID* | NA | 0.923 | 0.245 | 0.871 | 0.600 | 0.899 | 0.388 | 0.270 |
| *AGT M235T* | 0.965 | NA | 0.760 | 0.758 | 0.947 | 0.401 | 0.893 | 0.921 |
| *PAI- 1 4G5G* | 0.713 | 0.996 | NA | 0.914 | 0.887 | 0.286 | **0.049** | 0.646 |
| *REN G/T rs1464816* | 0.772 | 0.968 | 0.717 | NA | 0.444 | 0.991 | 0.836 | 0.689 |
| ETNK2 A/G  *rs1917542* | 0.545 | 0.819 | 0.893 | 0.759 | NA | NA | **0.006** | 0.928 |
| ETNK2 C/T  *rs2293337* | 0.836 | NA | **0.027** | 0.591 | **0.046** | NA | **0.034** | 0.930 |
| REN T/C  *rs3730103* | 0.409 | 0.906 | 0.309 | 0.607 | **0.030** | 0.135 | NA | 0.177 |
| *t-PA ID* | 0.405 | 0.733 | 0.828 | 0.493 | 0.714 | 0.970 | 0.593 | NA |
|  |  |  |  |  |  |  |  |  |
| **DxR** | *ACE ID* | AGT  *M235T* | *PAI-1 4G5G* | REN G/T  *rs1464816* | ETNK2 A/G  *rs1917542* | ETNK2 C/T  *rs2293337* | REN T/C  *rs3730103* | *t-PA ID* |
| *ACE_ID* | NA | 0.726 | 0.178 | 0.842 | 0.650 | 0.685 | 0.898 | 0.182 |
| *AGT M235T* | 0.795 | NA | 0.748 | 0.496 | 0.814 | 0.405 | 0.957 | 0.908 |
| *PAI- 1 4G5G* | 0.529 | 0.995 | NA | 0.703 | 0.875 | 0.288 | **0.020** | 0.374 |
| *REN G/T rs1464816* | 0.550 | 0.805 | 0.433 | NA | 0.227 | 0.999 | 0.830 | 0.893 |
| ETNK2 A/G  *rs1917542* | 0.504 | 0.686 | 0.888 | 0.716 | NA | NA | 0.346 | 0.828 |
| ETNK2 C/T  *rs2293337* | 0.577 | NA | **0.027** | 0.584 | **0.061** | NA | 0.280 | 0.811 |
| REN T/C  *rs3730103* | 0.700 | 0.919 | 0.889 | 0.322 | 0.482 | 0.205 | NA | **0.090** |
| *t-PA ID* | 0.934 | 0.707 | 0.695 | 0.578 | 0.621 | 0.972 | 0.420 | NA |

| **DxD** | *ACE ID* | AGT  *M235T* | *PAI-1 4G5G* | REN G/T  *rs1464816* | ETNK2 A/G  *rs1917542* | ETNK2 C/T  *rs2293337* | REN T/C  *rs3730103* | *t-PA ID* |
| --- | --- | --- | --- | --- | --- | --- | --- | --- |
| *ACE_ID* | NA | 0.984 | 0.821 | 0.626 | 0.492 | 0.713 | 0.176 | 0.195 |
| *AGT M235T* | 0.984 | NA | NA | 0.971 | 0.854 | NA | 0.640 | NA |
| *PAI4G5G* | 0.821 | NA | NA | 0.966 | 0.710 | NA | 0.137 | 0.547 |
| *REN G/T rs1464816* | 0.626 | 0.971 | 0.966 | NA | 0.583 | NA | 0.559 | 0.421 |
| ETNK2 A/G  *rs1917542* | 0.492 | 0.854 | 0.710 | 0.583 | NA | NA | **0.009** | 0.713 |
| ETNK2 C/T  *rs2293337* | 0.713 | NA | NA | NA | NA | NA | **0.055** | 0.828 |
| REN T/C  *rs3730103* | 0.176 | 0.640 | 0.137 | 0.559 | **0.009** | **0.055** | NA | 0.342 |
| *t-PA ID* | 0.195 | NA | 0.547 | 0.421 | 0.713 | 0.828 | 0.342 | NA |
|  |  |  |  |  |  |  |  |  |
| **RxA** | *ACE ID* | AGT  *M235T* | *PAI-1 4G5G* | REN G/T  *rs1464816* | ETNK2 A/G  *rs1917542* | ETNK2 C/T  *rs2293337* | REN T/C  *rs3730103* | *t-PA ID* |
| *ACE_ID* | NA | 0.784 | 0.488 | 0.531 | 0.740 | 0.829 | 0.168 | 0.839 |
| *AGT M235T* | 0.795 | NA | 0.843 | 0.917 | 0.904 | 0.623 | 0.801 | 0.369 |
| *PAI4G5G* | 0.295 | 0.721 | NA | 0.198 | 0.932 | **0.071** | 0.621 | 0.118 |
| *REN G/T rs1464816* | 0.544 | 0.773 | 0.198 | NA | 0.751 | 0.876 | 0.620 | 0.814 |
| ETNK2 A/G  *rs1917542* | 0.790 | 0.968 | 0.902 | 0.441 | NA | **0.065** | **0.085** | 0.873 |
| ETNK2 C/T  *rs2293337* | 0.912 | 0.683 | 0.342 | 0.977 | 0.413 | NA | 0.389 | 0.550 |
| REN T/C  *rs3730103* | 0.340 | 0.812 | **0.017** | 0.963 | 0.193 | 0.594 | NA | 0.552 |
| *t-PA ID* | 0.396 | 0.338 | **0.047** | 0.989 | 0.880 | 0.559 | 0.211 | NA |
|  |  |  |  |  |  |  |  |  |
| **RxR** | *ACE ID* | AGT  *M235T* | *PAI-1 4G5G* | REN G/T  *rs1464816* | ETNK2 A/G  *rs1917542* | ETNK2 C/T  *rs2293337* | REN T/C  *rs3730103* | *t-PA ID* |
| *ACE_ID* | NA | 0.623 | 0.239 | 0.270 | 0.513 | 0.909 | 0.112 | 0.560 |
| *AGT M235T* | 0.623 | NA | 0.569 | 0.792 | 0.801 | 0.654 | 0.544 | 0.168 |
| *PAI4G5G* | 0.239 | 0.569 | NA | **0.075** | 0.707 | 0.216 | 0.383 | **0.071** |
| *REN G/T rs1464816* | 0.270 | 0.792 | 0.075 | NA | 0.552 | 0.828 | 0.818 | 0.888 |
| ETNK2 A/G  *rs1917542* | 0.513 | 0.801 | 0.707 | 0.552 | NA | 0.406 | **0.064** | 0.755 |
| ETNK2 C/T  *rs2293337* | 0.909 | 0.654 | 0.216 | 0.828 | 0.406 | NA | 0.766 | 0.288 |
| REN T/C  *rs3730103* | 0.112 | 0.544 | 0.383 | 0.818 | **0.064** | 0.766 | NA | 0.343 |
| *t-PA ID* | 0.560 | 0.168 | **0.071** | 0.888 | 0.755 | 0.288 | 0.343 | NA |
|  |  |  |  |  |  |  |  |  |
| **RxD** | *ACE ID* | AGT  *M235T* | *PAI-1 4G5G* | REN G/T  *rs1464816* | ETNK2 A/G  *rs1917542* | ETNK2 C/T  *rs2293337* | REN T/C  *rs3730103* | *t-PA ID* |
| *ACE_ID* | NA | 0.795 | 0.529 | 0.550 | 0.504 | 0.577 | 0.700 | 0.934 |
| *AGT M235T* | 0.726 | NA | 0.995 | 0.805 | 0.686 | NA | 0.919 | 0.707 |
| *PAI4G5G* | 0.178 | 0.748 | NA | 0.433 | 0.888 | **0.027** | 0.889 | 0.695 |
| *REN G/T rs1464816* | 0.842 | 0.496 | 0.703 | NA | 0.716 | 0.584 | 0.322 | 0.578 |
| ETNK2 A/G  *rs1917542* | 0.650 | 0.814 | 0.875 | 0.227 | NA | **0.061** | 0.482 | 0.621 |
| ETNK2 C/T  *rs2293337* | 0.685 | 0.405 | 0.288 | 0.999 | NA | NA | 0.205 | 0.972 |
| REN T/C  *rs3730103* | 0.898 | 0.957 | **0.020** | 0.830 | 0.346 | 0.280 | NA | 0.420 |
| *t-PA ID* | 0.182 | 0.908 | 0.374 | 0.893 | 0.828 | 0.811 | **0.090** | NA |
|  |  |  |  |  |  |  |  |  |
| **AxA** | *ACE ID* | AGT  *M235T* | *PAI-1 4G5G* | REN G/T  *rs1464816* | ETNK2 A/G  *rs1917542* | ETNK2 C/T  *rs2293337* | REN T/C  *rs3730103* | *t-PA ID* |
| *ACE_ID* | NA | 0.942 | 0.442 | 0.820 | 0.784 | 0.971 | 0.358 | 0.579 |
| *AGT M235T* | 0.942 | NA | 0.881 | 0.954 | 0.976 | 0.670 | 0.960 | 0.527 |
| *PAI4G5G* | 0.442 | 0.881 | NA | 0.500 | 0.979 | 0.104 | **0.066** | 0.141 |
| *REN G/T rs1464816* | 0.820 | 0.954 | 0.500 | NA | 0.726 | 0.967 | 0.912 | 0.801 |
| ETNK2 A/G  *rs1917542* | 0.784 | 0.976 | 0.979 | 0.726 | NA | **0.052** | **0.011** | 0.945 |
| ETNK2 C/T  *rs2293337* | 0.971 | 0.670 | 0.104 | 0.967 | **0.052** | NA | 0.145 | 0.865 |
| REN T/C  *rs3730103* | 0.358 | 0.960 | **0.066** | 0.912 | **0.011** | 0.145 | NA | 0.446 |
| *t-PA ID* | 0.579 | 0.527 | 0.141 | 0.801 | 0.945 | 0.865 | 0.446 | NA |
|  |  |  |  |  |  |  |  |  |
| **AxD** | *ACE ID* | AGT  *M235T* | *PAI-1 4G5G* | REN G/T  *rs1464816* | ETNK2 A/G  *rs1917542* | ETNK2 C/T  *rs2293337* | REN T/C  *rs3730103* | *t-PA ID* |
| *ACE_ID* | NA | 0.965 | 0.713 | 0.772 | 0.545 | 0.836 | 0.409 | 0.405 |
| *AGT M235T* | 0.923 | NA | 0.996 | 0.968 | 0.819 | NA | 0.906 | 0.733 |
| *PAI4G5G* | 0.245 | 0.760 | NA | 0.717 | 0.893 | **0.027** | 0.309 | 0.828 |
| *REN G/T rs1464816* | 0.871 | 0.758 | 0.914 | NA | 0.759 | 0.591 | 0.607 | 0.493 |
| ETNK2 A/G  *rs1917542* | 0.600 | 0.947 | 0.887 | 0.444 | NA | **0.046** | **0.030** | 0.714 |
| ETNK2 C/T  *rs2293337* | 0.899 | 0.401 | 0.286 | 0.991 | NA | NA | 0.135 | 0.970 |
| REN T/C  *rs3730103* | 0.388 | 0.893 | **0.049** | 0.836 | **0.006** | **0.034** | NA | 0.593 |
| *t-PA ID* | 0.270 | 0.921 | 0.646 | 0.689 | 0.928 | 0.930 | 0.177 | NA |
|  |  |  |  |  |  |  |  |  |
| **AxR** | *ACE ID* | AGT  *M235T* | *PAI-1 4G5G* | REN G/T  *rs1464816* | ETNK2 A/G  *rs1917542* | ETNK2 C/T  *rs2293337* | REN T/C  *rs3730103* | *t-PA ID* |
| *ACE_ID* | NA | 0.795 | 0.295 | 0.544 | 0.790 | 0.912 | 0.340 | 0.396 |
| *AGT M235T* | 0.784 | NA | 0.721 | 0.773 | 0.968 | 0.683 | 0.812 | 0.338 |
| *PAI4G5G* | 0.488 | 0.843 | NA | 0.198 | 0.902 | 0.342 | **0.017** | **0.047** |
| *REN G/T rs1464816* | 0.531 | 0.917 | 0.198 | NA | 0.441 | 0.977 | 0.963 | 0.989 |
| ETNK2 A/G  *rs1917542* | 0.740 | 0.904 | 0.932 | 0.751 | NA | 0.413 | 0.193 | 0.880 |
| ETNK2 C/T  *rs2293337* | 0.829 | 0.623 | **0.071** | 0.876 | **0.065** | NA | 0.594 | 0.559 |
| REN T/C  *rs3730103* | 0.168 | 0.801 | 0.621 | 0.620 | **0.085** | 0.389 | NA | 0.211 |
| *t-PA ID* | 0.839 | 0.369 | 0.118 | 0.814 | 0.873 | 0.550 | 0.552 | NA |
