# Supplementary material for: Epistatic Interactions in Genetic Regulation of t-PA and PAI-1 Levels in a Ghanaian Population
Source: PLoS One. 2011 Jan 31;6(1):e16639. doi: 10.1371/journal.pone.0016639 (PMC3031598; doi:10.1371/journal.pone.0016639)
Supplement: Table S2 — A. Variability (r2) of t-PA and PAI-1 levels explained by models combining two SNPs without an interaction term (SNP1 + SNP2), models combining two SNPs with an interaction term (SNP1*SNP2) and the variability obtained by extracting only the interaction term from the full model in females. Results shown meet our exploratory statistical threshold of p < 0.10 for the interaction term from the full model. * denote single SNP analysis (see Schoenhard et al. 2008 [16]). D = dominant encoding, A = additive encoding, R = recessive encoding. (DOC) [file pone.0016639.s002.doc]

**Table S2.**

| **Female tPA** | **SNP1** | **SNP2** | | **SNP1 + SNP2 model R2*100** | | **SNP1 + SNP2 model p-value** | | **SNP1 * SNP2 model R2*100** | **SNP1 * SNP2 model p-value** | **SNP1 * SNP2 interaction term R2*100** | **SNP1 * SNP2 interaction term p-value** |
| --- | --- | --- | --- | --- | --- | --- | --- | --- | --- | --- | --- |
|  | REN G6567T (rs1464816) D | NA | | 0.700* | | 0.049* | | NA | NA | NA | NA |
|  | REN T9435C (rs3730103) A | ETNK2 A6135G (rs1917542) A | | 0.631 | | 0.465 | | 2.877 | 0.036 | 2.270 | 0.011 |
|  | REN T9435C (rs3730103) D | ETNK2 A6135G (rs1917542) A | | 0.326 | | 0.604 | | 2.148 | 0.031 | 1.820 | 0.006 |
|  | REN T9435C (rs3730103) A | PAI 4G/5G (rs1799768) A | | 0.649 | | 0.450 | | 2.176 | 0.134 | 1.540 | 0.066 |
|  | PAI 4G/5G (rs1799768) R | REN T9435C (rs3730103) A | | 0.649 | | 0.297 | | 2.056 | 0.038 | 1.420 | 0.017 |
|  | ETNK2 A6135G (rs1917542) D | REN T9435C (rs3730103) A | | 0.627 | | 0.313 | | 1.848 | 0.061 | 1.220 | 0.030 |
|  | REN T9435C (rs3730103) D | ETNK2 C5047T (rs2293337) A | | 0.577 | | 0.351 | | 1.769 | 0.073 | 1.190 | 0.034 |
|  | REN T9435C (rs3730103) D | ETNK2 A6135G (rs1917542) D | | 0.318 | | 0.405 | | 1.501 | 0.036 | 1.180 | 0.009 |
|  | PAI 4G/5G (rs1799768) R | TPA I/D (rs4646972) A | | 0.526 | | 0.393 | | 1.622 | 0.100 | 1.080 | 0.047 |
|  | REN T9435C (rs3730103) D | PAI 4G/5G (rs1799768) A | | 0.371 | | 0.550 | | 1.431 | 0.148 | 1.060 | 0.049 |
|  | ETNK2 C5047T (rs2293337) A | ETNK2 A6135G (rs1917542) A | | 0.597 | | 0.495 | | 1.662 | 0.149 | 1.040 | 0.052 |
|  | REN T9435C (rs3730103) D | PAI 4G/5G (rs1799768) R | | 0.370 | | 0.349 | | 1.326 | 0.056 | 0.960 | 0.020 |
|  | ETNK2 C5047T (rs2293337) R | ETNK2 A6135G (rs1917542) A | | 0.596 | | 0.336 | | 1.583 | 0.108 | 0.960 | 0.065 |
|  | ETNK2 C5047T (rs2293337) R | PAI 4G/5G (rs1799768) A | | 0.640 | | 0.303 | | 1.559 | 0.114 | 0.930 | 0.071 |
|  | REN T9435C (rs3730103) R | ETNK2 A6135G (rs1917542) A | | 0.372 | | 0.549 | | 1.218 | 0.226 | 0.890 | 0.085 |
|  | PAI 4G/5G (rs1799768) D | ETNK2 C5047T (rs2293337) A | | 0.484 | | 0.432 | | 1.337 | 0.107 | 0.860 | 0.027 |
|  | PAI 4G/5G (rs1799768) D | ETNK2 C5047T (rs2293337) R | | 0.479 | | 0.256 | | 1.337 | 0.054 | 0.860 | 0.027 |
|  | ETNK2 A6135G (rs1917542) D | ETNK2 C5047T (rs2293337) A | | 0.533 | | 0.387 | | 1.221 | 0.138 | 0.700 | 0.046 |
|  | REN T9435C (rs3730103) D | ETNK2 C5047T (rs2293337) D | | 0.216 | | 0.542 | | 0.864 | 0.178 | 0.650 | 0.055 |
|  | ETNK2 A6135G (rs1917542) D | ETNK2 C5047T (rs2293337) R | | 0.533 | | 0.220 | | 1.141 | 0.090 | 0.620 | 0.061 |
|  | REN T9435C (rs3730103) R | ETNK2 A6135G (rs1917542) R | | 0.158 | | 0.638 | | 0.740 | 0.240 | 0.610 | 0.064 |
|  | TPA I/D (rs4646972) R | PAI 4G/5G (rs1799768) R | | 0.310 | | 0.415 | | 0.896 | 0.165 | 0.570 | 0.071 |
|  | REN G6567T (rs1464816) R | PAI 4G/5G (rs1799768) R | | 0.992 | | 0.059 | | 1.560 | 0.031 | 0.550 | 0.075 |
|  | TPA I/D (rs4646972) D | REN T9435C (rs3730103) R | | 0.237 | | 0.510 | | 0.749 | 0.235 | 0.510 | 0.090 |
|  |  | |  | |  | |  |  |  |  |  |
|  |  | |  | |  | |  |  |  |  |  |
| **Female PAI-1** | **SNP1** | **SNP2** | | **SNP1 + SNP2 model R2*100** | | **SNP1 + SNP2 model p-value** | | **SNP1 * SNP2 model R2*100** | **SNP1 * SNP2 model p-value** | **SNP1 * SNP2 interaction term R2*100** | **SNP1 * SNP2 interaction term p-value** |
|  | PAI 4G/5G (rs1799768) A | NA | | 0.800* | | 0.029* | | NA | NA | NA | NA |
|  | TPA I/D (rs4646972) A | REN T9435C (rs3730103) A | | 0.609 | | 0.485 | | 3.028 | 0.027 | 2.420 | 0.008 |
|  | TPA I/D (rs4646972) A | REN G6567T (rs1464816) A | | 0.255 | | 0.836 | | 2.311 | 0.106 | 2.050 | 0.020 |
|  | TPA I/D (rs4646972) D | REN T9435C (rs3730103) A | | 0.581 | | 0.347 | | 2.565 | 0.012 | 1.980 | 0.003 |
|  | TPA I/D (rs4646972) D | REN T9435C (rs3730103) D | | 0.519 | | 0.229 | | 2.497 | 0.002 | 1.980 | 0.001 |
|  | REN G6567T (rs1464816) D | TPA I/D (rs4646972) A | | 0.233 | | 0.724 | | 1.917 | 0.052 | 1.670 | 0.008 |
|  | REN T9435C (rs3730103) A | ACE I/D (rs4646994) A | | 0.473 | | 0.612 | | 2.114 | 0.149 | 1.640 | 0.053 |
|  | TPA I/D (rs4646972) D | REN G6567T (rs1464816) D | | 0.198 | | 0.571 | | 1.732 | 0.019 | 1.530 | 0.003 |
|  | REN G6567T (rs1464816) A | AGT M235T (rs699) A | | 0.237 | | 0.854 | | 1.726 | 0.278 | 1.470 | 0.080 |
|  | REN G6567T (rs1464816) D | AGT M235T (rs699) A | | 0.205 | | 0.762 | | 1.619 | 0.100 | 1.390 | 0.019 |
|  | ACE I/D (rs4646994) R | REN T9435C (rs3730103) A | | 0.385 | | 0.534 | | 1.612 | 0.102 | 1.230 | 0.030 |
|  | REN T9435C (rs3730103) D | ACE I/D (rs4646994) A | | 0.434 | | 0.482 | | 1.601 | 0.104 | 1.170 | 0.036 |
|  | PAI 4G/5G (rs1799768) R | REN G6567T (rs1464816) A | | 0.675 | | 0.280 | | 1.632 | 0.097 | 0.960 | 0.065 |
|  | REN T9435C (rs3730103) D | ACE I/D (rs4646994) R | | 0.348 | | 0.372 | | 1.285 | 0.062 | 0.940 | 0.021 |
|  | REN T9435C (rs3730103) D | PAI 4G/5G (rs1799768) A | | 1.040 | | 0.115 | | 1.856 | 0.060 | 0.820 | 0.097 |
|  | REN T9435C (rs3730103) R | ACE I/D (rs4646994) R | | 0.100 | | 0.752 | | 0.740 | 0.240 | 0.640 | 0.057 |
|  | TPA I/D (rs4646972) R | REN T9435C (rs3730103) R | | 0.109 | | 0.734 | | 0.634 | 0.307 | 0.530 | 0.084 |
|  | AGT M235T (rs699) D | ACE I/D (rs4646994) R | | 0.256 | | 0.483 | | 0.648 | 0.298 | 0.520 | 0.087 |
|  | REN G6567T (rs1464816) D | AGT M235T (rs699) D | | 0.161 | | 0.633 | | 0.706 | 0.260 | 0.520 | 0.087 |
|  | REN T9435C (rs3730103) D | PAI 4G/5G (rs1799768) D | | 0.654 | | 0.156 | | 1.169 | 0.084 | 0.520 | 0.086 |
|  | REN G6567T (rs1464816) R | PAI 4G/5G (rs1799768) R | | 0.670 | | 0.149 | | 1.180 | 0.081 | 0.510 | 0.087 |
|  | ETNK2 A6135G (rs1917542) R | REN G6567T (rs1464816) R | | 0.154 | | 0.646 | | 0.652 | 0.295 | 0.510 | 0.090 |

B. Variability (r^2^) of t-PA and PAI-1 levels explained by models combining two SNPs without an interaction term (SNP1 + SNP2), models combining two SNPs with an interaction term (SNP1*SNP2) and the variability obtained by extracting only the interaction term from the full model in males. Results shown meet our exploratory statistical threshold of p < 0.10 for the interaction term from the full model. * denote single SNP analysis (see Schoenhard 2008). D= dominant encoding, A = additive encoding, R = recessive encoding.

| **Male tPA** | **SNP1** | **SNP2** | **SNP1 + SNP2 model R2*100** | **SNP1 + SNP2 model p-value** | **SNP1 * SNP2 model R2*100** | **SNP1 * SNP2 model p-value** | **SNP1 * SNP2 interaction term R2*100** | **SNP1 * SNP2 interaction term p-value** |
| --- | --- | --- | --- | --- | --- | --- | --- | --- |
|  | TPA I/D (rs4646972) A | NA | 1.200* | 0.026* | NA | NA | NA | NA |
|  | REN T9435C (rs3730103) A | NA | 1.300* | 0.019* | NA | NA | NA | NA |
|  | TPA I/D (rs4646972) A | PAI 4G/5G (rs1799768) A | 1.480 | 0.182 | 4.097 | 0.026 | 2.620 | 0.025 |
|  | REN G6567T (rs1464816) A | AGT M235T (rs699) A | 0.988 | 0.386 | 3.020 | 0.078 | 2.030 | 0.035 |
|  | TPA I/D (rs4646972) A | REN T9435C (rs3730103) A | 2.613 | 0.026 | 4.614 | 0.012 | 2.000 | 0.072 |
|  | REN T9435C (rs3730103) R | TPA I/D (rs4646972) A | 2.528 | 0.013 | 4.446 | 0.002 | 1.920 | 0.016 |
|  | ETNK2 A6135G (rs1917542) A | PAI 4G/5G (rs1799768) A | 0.396 | 0.798 | 2.305 | 0.287 | 1.910 | 0.091 |
|  | TPA I/D (rs4646972) D | REN T9435C (rs3730103) A | 2.518 | 0.014 | 4.373 | 0.002 | 1.850 | 0.018 |
|  | TPA I/D (rs4646972) D | REN T9435C (rs3730103) R | 0.149 | 0.655 | 0.177 | 0.801 | 1.800 | 0.005 |
|  | AGT M235T (rs699) R | REN G6567T (rs1464816) A | 0.852 | 0.310 | 2.556 | 0.055 | 1.700 | 0.027 |
|  | ETNK2 A6135G (rs1917542) R | PAI 4G/5G (rs1799768) A | 0.256 | 0.784 | 1.956 | 0.143 | 1.700 | 0.028 |
|  | REN G6567T (rs1464816) D | AGT M235T (rs699) R | 0.132 | 0.687 | 0.212 | 0.753 | 1.650 | 0.008 |
|  | ACE I/D (rs4646994) R | TPA I/D (rs4646972) A | 1.458 | 0.105 | 3.085 | 0.023 | 1.630 | 0.031 |
|  | REN G6567T (rs1464816) D | AGT M235T (rs699) A | 0.413 | 0.629 | 1.981 | 0.079 | 1.570 | 0.010 |
|  | PAI 4G/5G (rs1799768) R | TPA I/D (rs4646972) A | 1.451 | 0.106 | 2.907 | 0.031 | 1.460 | 0.045 |
|  | TPA I/D (rs4646972) D | PAI 4G/5G (rs1799768) A | 1.335 | 0.131 | 2.705 | 0.043 | 1.370 | 0.055 |
|  | ETNK2 A6135G (rs1917542) D | ACE I/D (rs4646994) A | 0.408 | 0.635 | 1.738 | 0.198 | 1.330 | 0.061 |
|  | PAI 4G/5G (rs1799768) D | ETNK2 A6135G (rs1917542) A | 0.396 | 0.646 | 1.588 | 0.245 | 1.190 | 0.082 |
|  | PAI 4G/5G (rs1799768) D | ETNK2 A6135G (rs1917542) R | 0.016 | 0.956 | 0.019 | 0.991 | 1.120 | 0.030 |
|  | ACE I/D (rs4646994) R | ETNK2 A6135G (rs1917542) A | 0.356 | 0.684 | 1.477 | 0.286 | 1.120 | 0.095 |
|  | ETNK2 A6135G (rs1917542) D | ACE I/D (rs4646994) R | 0.382 | 0.338 | 0.418 | 0.499 | 1.090 | 0.032 |
|  | TPA I/D (rs4646972) D | ACE I/D (rs4646994) r | 0.297 | 0.430 | 0.611 | 0.325 | 1.030 | 0.037 |
|  | ETNK2 A6135G (rs1917542) R | PAI 4G/5G (rs1799768) R | 0.212 | 0.641 | 1.232 | 0.159 | 1.020 | 0.038 |
|  | TPA I/D (rs4646972) R | ACE I/D (rs4646994) R | 0.478 | 0.367 | 1.456 | 0.105 | 0.980 | 0.042 |
|  | TPA I/D (rs4646972) D | PAI 4G/5G (rs1799768) R | 0.269 | 0.466 | 0.409 | 0.509 | 0.890 | 0.052 |
|  | ETNK2 A6135G (rs1917542) R | AGT M235T (rs699) R | 0.258 | 0.582 | 1.054 | 0.218 | 0.800 | 0.067 |
|  | REN G6567T (rs1464816) D | ETNK2 A6135G (rs1917542) R | 0.039 | 0.896 | 0.058 | 0.955 | 0.790 | 0.067 |
|  | TPA I/D (rs4646972) R | REN G6567T (rs1464816) R | 1.152 | 0.088 | 1.910 | 0.045 | 0.760 | 0.073 |
|  | ETNK2 A6135G (rs1917542) D | ETNK2 C5047T (rs2293337) R | 0.533 | 0.220 | 1.141 | 0.090 | 0.720 | 0.081 |
|  | ETNK2 A6135G (rs1917542) R | AGT M235T (rs699) A | 0.256 | 0.784 | 1.956 | 0.143 | 0.700 | 0.086 |
|  | ETNK2 A6135G (rs1917542) D | ETNK2 C5047T (rs2293337) A | 0.310 | 0.729 | 0.989 | 0.386 | 0.680 | 0.092 |
|  | ACE I/D (rs4646994) D | ETNK2 C5047T (rs2293337) R | 0.466 | 0.266 | 0.523 | 0.396 | 0.680 | 0.090 |

| **Male PAI-1** | **SNP1** | **SNP2** | **SNP1 + SNP2 model R2*100** | **SNP1 + SNP2 model p-value** | **SNP1 * SNP2 model R2*100** | **SNP1 * SNP2 model p-value** | **SNP1 * SNP2 interaction term R2*100** | **SNP1 * SNP2 interaction term p-value** |
| --- | --- | --- | --- | --- | --- | --- | --- | --- |
|  | REN G6567T (rs1464816) D | NA | 0.900* | 0.046* | NA | NA | NA | NA |
|  | ETNK2 A6135G (rs1917542) A | NA | 0.800* | 0.066* | NA | NA | NA | NA |
|  | REN T9435C (rs3730103) R | NA | 1.000* | 0.040* | NA | NA | NA | NA |
|  | TPA I/D (rs4646972) R | NA | 1.000* | 0.044* | NA | NA | NA | NA |
|  | REN G6567T (rs1464816) R | ETNK2 A6135G (rs1917542) A | 1.541 | 0.090 | 2.849 | 0.034 | 1.310 | 0.062 |
|  | TPA I/D (rs4646972) R | REN G6567T (rs1464816) A | 1.071 | 0.212 | 2.251 | 0.090 | 1.180 | 0.082 |
|  | REN G6567T (rs1464816) R | TPA I/D (rs4646972) A | 2.089 | 0.032 | 3.176 | 0.019 | 1.090 | 0.098 |
|  | TPA I/D (rs4646972) R | REN G6567T (rs1464816) R | 1.047 | 0.110 | 2.125 | 0.029 | 1.080 | 0.032 |
|  | ETNK2 A6135G (rs1917542) D | ACE I/D (rs4646994) R | 0.803 | 0.185 | 1.652 | 0.073 | 0.850 | 0.058 |
|  | ETNK2 A6135G (rs1917542) R | REN G6567T (rs1464816) R | 1.237 | 0.074 | 2.056 | 0.034 | 0.820 | 0.062 |
|  | PAI 4G/5G (rs1799768) D | ETNK2 A6135G (rs1917542) R | 0.831 | 0.174 | 1.509 | 0.095 | 0.680 | 0.091 |
