# Supplementary material for: Epistatic Interactions in Genetic Regulation of t-PA and PAI-1 Levels in a Ghanaian Population
Source: PLoS One. 2011 Jan 31;6(1):e16639. doi: 10.1371/journal.pone.0016639 (PMC3031598; doi:10.1371/journal.pone.0016639)
Supplement: Table S3 — p -values for epistatic effects between polymorphisms in association with plasma PAI-1 levels for females. All nine interaction models are presented. Each table displays the results of one interaction pairing as indicated in the upper left-hand corner. The notation is column by row, for example, a DxA interaction indicates that the SNPs across the columns are encoded as dominant while the SNPs down the rows are endoded as additive. p-values <0.10 are displayed in boldface. D = dominant, A = additive, R = recessive. (DOC) [file pone.0016639.s003.doc]

**Supplemental Table 3.**

| **DxA** | *ACE_ID* | AGT  *M235T* | *PAI4G5G* | REN G/T  *rs1464816* | ETNK2 A/G  *rs1917542* | ETNK2 C/T  *rs2293337* | REN T/C  *rs3730103* | *t-PA ID* |
| --- | --- | --- | --- | --- | --- | --- | --- | --- |
| *ACE_ID* | NA | 0.120 | 0.758 | 0.239 | 0.735 | 0.883 | **0.036** | 0.299 |
| *AGT M235T* | 0.702 | NA | 0.963 | **0.019** | 0.577 | 0.746 | 0.685 | 0.511 |
| *PAI- 1 4G5G* | 0.517 | 0.851 | NA | 0.334 | 0.400 | 0.349 | **0.097** | 0.116 |
| *REN G/T rs1464816* | 0.155 | 0.201 | 0.769 | NA | 0.367 | 0.648 | 0.419 | **0.009** |
| ETNK2 A/G  *rs1917542* | 0.582 | 0.565 | 0.842 | 0.591 | NA | NA | 0.159 | 0.452 |
| ETNK2 C/T  *rs2293337* | 0.855 | NA | 0.317 | 0.331 | 0.153 | NA | 0.889 | 0.909 |
| REN T/C  *rs3730103* | 0.309 | 0.780 | 0.187 | 0.338 | 0.293 | 0.827 | NA | **0.003** |
| *t-PA ID* | 0.286 | 0.675 | 0.994 | **0.008** | 0.179 | 0.814 | **0.003** | NA |
|  |  |  |  |  |  |  |  |  |
| **DxR** | *ACE_ID* | AGT  *M235T* | *PAI4G5G* | REN G/T  *rs1464816* | ETNK2 A/G  *rs1917542* | ETNK2 C/T  *rs2293337* | REN T/C  *rs3730103* | *t-PA ID* |
| *ACE_ID* | NA | **0.087** | 0.496 | 0.324 | 0.687 | 0.771 | **0.021** | 0.446 |
| *AGT M235T* | 0.414 | NA | 0.906 | 0.129 | 0.441 | 0.772 | 0.437 | 0.571 |
| *PAI- 1 4G5G* | 0.320 | 0.874 | NA | 0.306 | 0.176 | 0.376 | 0.491 | 0.043 |
| *REN G/T rs1464816* | 0.157 | 0.336 | 0.540 | NA | 0.224 | 0.640 | 0.406 | 0.972 |
| ETNK2 A/G  *rs1917542* | 0.454 | 0.994 | 0.695 | 0.986 | NA | NA | 0.633 | 0.524 |
| ETNK2 C/T  *rs2293337* | 0.907 | NA | 0.329 | 0.319 | 0.191 | NA | 0.604 | 0.778 |
| REN T/C  *rs3730103* | 0.238 | 0.698 | 0.319 | 0.468 | 0.517 | 0.643 | NA | 0.385 |
| *t-PA ID* | 0.383 | 0.656 | 0.990 | 0.933 | 0.420 | 0.639 | 0.165 | NA |
|  |  |  |  |  |  |  |  |  |
| **DxD** | *ACE_ID* | AGT  *M235T* | *PAI4G5G* | REN G/T  *rs1464816* | ETNK2 A/G  *rs1917542* | ETNK2 C/T  *rs2293337* | REN T/C  *rs3730103* | *t-PA ID* |
| *ACE_ID* | NA | 0.739 | 0.965 | 0.106 | 0.627 | 0.619 | 0.551 | 0.118 |
| *AGT M235T* | 0.739 | NA | NA | **0.087** | 0.393 | NA | 0.495 | NA |
| *PAI-1 4G5G* | 0.965 | NA | NA | 0.570 | 0.771 | NA | **0.086** | 0.935 |
| *REN G/T rs1464816* | 0.106 | 0.087 | 0.570 | NA | 0.340 | NA | 0.244 | **0.003** |
| ETNK2 A/G  *rs1917542* | 0.627 | 0.393 | 0.771 | 0.340 | NA | NA | 0.129 | 0.218 |
| ETNK2 C/T  *rs2293337* | 0.619 | NA | NA | NA | NA | NA | 0.716 | 0.807 |
| REN T/C  *rs3730103* | 0.551 | 0.495 | 0.086 | 0.244 | 0.129 | 0.716 | NA | **0.001** |
| *t-PA ID* | 0.118 | NA | 0.935 | 0.003 | 0.218 | 0.807 | 0.001 | NA |
|  |  |  |  |  |  |  |  |  |
| **RxA** | *ACE_ID* | AGT  *M235T* | *PAI4G5G* | REN G/T  *rs1464816* | ETNK2 A/G  *rs1917542* | ETNK2 C/T  *rs2293337* | REN T/C  *rs3730103* | *t-PA ID* |
| *ACE_ID* | NA | 0.227 | 0.334 | 0.320 | 0.265 | 0.951 | 0.144 | 0.405 |
| *AGT M235T* | 0.145 | NA | 0.697 | 0.397 | 0.997 | 0.151 | 0.707 | 0.304 |
| *PAI-1 4G5G* | 0.189 | 0.627 | NA | 0.204 | 0.340 | 0.517 | 0.474 | 0.934 |
| *REN G/T rs1464816* | 0.517 | 0.298 | **0.065** | NA | 0.207 | 0.469 | 0.548 | 0.386 |
| ETNK2 A/G  *rs1917542* | 0.252 | 0.740 | 0.286 | 0.187 | NA | 0.348 | 0.616 | 0.535 |
| ETNK2 C/T  *rs2293337* | 0.874 | 0.445 | 0.561 | 0.838 | 0.577 | NA | 0.900 | 0.576 |
| REN T/C  *rs3730103* | **0.030** | 0.535 | 0.781 | 0.301 | 0.876 | 0.853 | NA | 0.147 |
| *t-PA ID* | 0.429 | 0.364 | 0.121 | 0.398 | 0.355 | 0.587 | 0.209 | NA |
|  |  |  |  |  |  |  |  |  |
| **RxR** | *ACE_ID* | AGT  *M235T* | *PAI4G5G* | REN G/T  *rs1464816* | ETNK2 A/G  *rs1917542* | ETNK2 C/T  *rs2293337* | REN T/C  *rs3730103* | *t-PA ID* |
| *ACE_ID* | NA | 0.205 | 0.168 | 0.375 | 0.100 | 0.760 | **0.057** | 0.203 |
| *AGT M235T* | 0.205 | NA | 0.449 | 0.486 | 0.859 | 0.201 | 0.576 | 0.154 |
| *PAI-1 4G5G* | 0.168 | 0.449 | NA | **0.087** | 0.231 | 0.744 | 0.759 | 0.716 |
| *REN G/T rs1464816* | 0.375 | 0.486 | 0.087 | NA | **0.090** | 0.659 | 0.314 | 0.196 |
| ETNK2 A/G  *rs1917542* | 0.100 | 0.859 | 0.231 | 0.090 | NA | 0.560 | 0.652 | 0.320 |
| ETNK2 C/T  *rs2293337* | 0.760 | 0.201 | 0.744 | 0.659 | 0.560 | NA | 0.851 | 0.300 |
| REN T/C  *rs3730103* | 0.057 | 0.576 | 0.759 | 0.314 | 0.652 | 0.851 | NA | **0.084** |
| *t-PA ID* | 0.203 | 0.154 | 0.716 | 0.196 | 0.320 | 0.300 | 0.084 | NA |
|  |  |  |  |  |  |  |  |  |
| **RxD** | *ACE_ID* | AGT  *M235T* | *PAI4G5G* | REN G/T  *rs1464816* | ETNK2 A/G  *rs1917542* | ETNK2 C/T  *rs2293337* | REN T/C  *rs3730103* | *t-PA ID* |
| *ACE_ID* | NA | 0.414 | 0.320 | 0.157 | 0.454 | 0.907 | 0.238 | 0.383 |
| *AGT M235T* | **0.087** | NA | 0.874 | 0.336 | 0.994 | NA | 0.698 | 0.656 |
| *PAI-1 4G5G* | 0.496 | 0.906 | NA | 0.540 | 0.695 | 0.329 | 0.319 | 0.990 |
| *REN G/T rs1464816* | 0.324 | 0.129 | 0.306 | NA | 0.986 | 0.319 | 0.468 | 0.933 |
| ETNK2 A/G  *rs1917542* | 0.687 | 0.441 | 0.176 | 0.224 | NA | 0.191 | 0.517 | 0.420 |
| ETNK2 C/T  *rs2293337* | 0.771 | 0.772 | 0.376 | 0.640 | NA | NA | 0.643 | 0.639 |
| REN T/C  *rs3730103* | **0.021** | 0.437 | 0.491 | 0.406 | 0.633 | 0.604 | NA | 0.165 |
| *t-PA ID* | 0.446 | 0.571 | 0.043 | 0.972 | 0.524 | 0.778 | 0.385 | NA |
|  |  |  |  |  |  |  |  |  |
| **AxA** | *ACE_ID* | AGT  *M235T* | *PAI4G5G* | REN G/T  *rs1464816* | ETNK2 A/G  *rs1917542* | ETNK2 C/T  *rs2293337* | REN T/C  *rs3730103* | *t-PA ID* |
| *ACE_ID* | NA | 0.182 | 0.440 | 0.391 | 0.524 | 0.978 | **0.053** | 0.474 |
| *AGT M235T* | 0.182 | NA | 0.783 | **0.080** | 0.819 | 0.360 | 0.803 | 0.497 |
| *PAI- 1 4G5G* | 0.440 | 0.783 | NA | 0.140 | 0.522 | 0.430 | 0.266 | 0.351 |
| *REN G/T rs1464816* | 0.391 | 0.080 | 0.140 | NA | 0.361 | 0.667 | 0.446 | **0.020** |
| ETNK2 A/G  *rs1917542* | 0.524 | 0.819 | 0.522 | 0.361 | NA | 0.296 | 0.417 | 0.365 |
| ETNK2 C/T  *rs2293337* | 0.978 | 0.360 | 0.430 | 0.667 | 0.296 | NA | 0.974 | 0.864 |
| REN T/C  *rs3730103* | 0.053 | 0.803 | 0.266 | 0.446 | 0.417 | 0.974 | NA | **0.008** |
| *t-PA ID* | 0.474 | 0.497 | 0.351 | 0.020 | 0.365 | 0.864 | 0.008 | NA |
|  |  |  |  |  |  |  |  |  |
| **AxD** | *ACE_ID* | AGT  *M235T* | *PAI4G5G* | REN G/T  *rs1464816* | ETNK2 A/G  *rs1917542* | ETNK2 C/T  *rs2293337* | REN T/C  *rs3730103* | *t-PA ID* |
| *ACE_ID* | NA | 0.702 | 0.517 | 0.155 | 0.582 | 0.855 | 0.309 | 0.286 |
| *AGT M235T* | 0.120 | NA | 0.851 | 0.201 | 0.565 | NA | 0.780 | 0.675 |
| *PAI-1 4G5G* | 0.758 | 0.963 | NA | 0.769 | 0.842 | 0.317 | 0.187 | 0.994 |
| *REN G/T rs1464816* | 0.239 | **0.019** | 0.334 | NA | 0.591 | 0.331 | 0.338 | **0.008** |
| ETNK2 A/G  *rs1917542* | 0.735 | 0.577 | 0.400 | 0.367 | NA | 0.153 | 0.293 | 0.179 |
| ETNK2 C/T  *rs2293337* | 0.883 | 0.746 | 0.349 | 0.648 | NA | NA | 0.827 | 0.814 |
| REN T/C  *rs3730103* | **0.036** | 0.685 | **0.097** | 0.419 | 0.159 | 0.889 | NA | **0.003** |
| *t-PA ID* | 0.299 | 0.511 | 0.116 | **0.009** | 0.452 | 0.909 | **0.003** | NA |
|  |  |  |  |  |  |  |  |  |
| **AxR** | *ACE_ID* | AGT  *M235T* | *PAI4G5G* | REN G/T  *rs1464816* | ETNK2 A/G  *rs1917542* | ETNK2 C/T  *rs2293337* | REN T/C  *rs3730103* | *t-PA ID* |
| *ACE_ID* | NA | 0.145 | 0.189 | 0.517 | 0.252 | 0.874 | **0.030** | 0.429 |
| *AGT M235T* | 0.227 | NA | 0.627 | 0.298 | 0.740 | 0.445 | 0.535 | 0.364 |
| *PAI-1 4G5G* | 0.334 | 0.697 | NA | **0.065** | 0.286 | 0.561 | 0.781 | 0.121 |
| *REN G/T rs1464816* | 0.320 | 0.397 | 0.204 | NA | 0.187 | 0.838 | 0.301 | 0.398 |
| ETNK2 A/G  *rs1917542* | 0.265 | 0.997 | 0.340 | 0.207 | NA | 0.577 | 0.876 | 0.355 |
| ETNK2 C/T  *rs2293337* | 0.951 | 0.151 | 0.517 | 0.469 | 0.348 | NA | 0.853 | 0.587 |
| REN T/C  *rs3730103* | 0.144 | 0.707 | 0.474 | 0.548 | 0.616 | 0.900 | NA | 0.209 |
| *t-PA ID* | 0.405 | 0.304 | 0.934 | 0.386 | 0.535 | 0.576 | 0.147 | NA |
