# Supplementary material for: Epistatic Interactions in Genetic Regulation of t-PA and PAI-1 Levels in a Ghanaian Population
Source: PLoS One. 2011 Jan 31;6(1):e16639. doi: 10.1371/journal.pone.0016639 (PMC3031598; doi:10.1371/journal.pone.0016639)
Supplement: Table S4 — p -values for epistatic effects between polymorphisms in association with plasma t-PA levels for males. All nine interaction models are presented. Each table displays the results of one interaction pairing as indicated in the upper left-hand corner. The notation is column by row, for example, a DxA interaction indicates that the SNPs across the columns are encoded as dominant while the SNPs down the rows are endoded as additive. p-values <0.10 are displayed in boldface. D = dominant, A = additive, R = recessive. (DOC) [file pone.0016639.s004.doc]

**Table S4**.

| **DxA** | *ACE ID* | AGT  *M235T* | *PAI-1 4G5G* | REN G/T  *rs1464816* | ETNK2 A/G  *rs1917542* | ETNK2 C/T  *rs2293337* | REN T/C  *rs3730103* | *t-PA ID* |
| --- | --- | --- | --- | --- | --- | --- | --- | --- |
| *ACE_ID* | NA | 0.467 | 0.441 | 0.131 | **0.061** | 0.813 | 0.327 | 0.113 |
| *AGT M235T* | 0.297 | NA | 0.603 | **0.010** | 0.869 | 0.984 | 0.436 | 0.867 |
| *PAI- 1 4G5G* | 0.640 | 0.654 | NA | 0.408 | 0.479 | 0.717 | 0.808 | **0.055** |
| *REN G/T rs1464816* | 0.236 | 0.240 | 0.312 | NA | 0.237 | 0.591 | 0.305 | 0.411 |
| ETNK2 A/G  *rs1917542* | 0.242 | 0.899 | **0.082** | 0.155 | NA | NA | 0.609 | 0.215 |
| ETNK2 C/T  *rs2293337* | 0.137 | 0.905 | 0.168 | 0.684 | **0.092** | NA | 0.751 | 0.962 |
| REN T/C  *rs3730103* | 0.670 | 0.568 | 0.928 | 0.415 | 0.613 | 0.717 | NA | **0.018** |
| *t-PA ID* | 0.587 | 0.655 | 0.342 | 0.659 | 0.788 | 0.933 | 0.287 | NA |
|  |  |  |  |  |  |  |  |  |
| **DxR** | *ACE ID* | AGT  *M235T* | *PAI-1 4G5G* | REN G/T  *rs1464816* | ETNK2 A/G  *rs1917542* | ETNK2 C/T  *rs2293337* | REN T/C  *rs3730103* | *t-PA ID* |
| *ACE_ID* | NA | 0.484 | 0.195 | 0.299 | **0.032** | 0.994 | 0.299 | **0.037** |
| *AGT M235T* | 0.350 | NA | 0.623 | **0.008** | 0.615 | 0.711 | 0.430 | 0.915 |
| *PAI- 1 4G5G* | 0.483 | 0.665 | NA | 0.443 | 0.254 | 0.483 | 0.723 | **0.052** |
| *REN G/T rs1464816* | 0.146 | 0.235 | 0.327 | NA | 0.247 | 0.357 | 0.482 | 0.235 |
| ETNK2 A/G  *rs1917542* | 0.854 | NA | **0.030** | **0.067** | NA | NA | 0.889 | 0.103 |
| ETNK2 C/T  *rs2293337* | **0.090** | 0.730 | 0.138 | 0.384 | **0.081** | NA | 0.524 | 0.934 |
| REN T/C  *rs3730103* | 0.691 | 0.527 | 0.822 | 0.680 | 0.583 | 0.632 | NA | **0.005** |
| *t-PA ID* | 0.342 | 0.617 | 0.465 | 0.343 | 0.472 | 0.731 | 0.921 | NA |

|  |  |  |  |  |  |  |  |  |
| --- | --- | --- | --- | --- | --- | --- | --- | --- |
| **DxD** | *ACE ID* | AGT  *M235T* | *PAI-1 4G5G* | REN G/T  *rs1464816* | ETNK2 A/G  *rs1917542* | ETNK2 C/T  *rs2293337* | REN T/C  *rs3730103* | *t-PA ID* |
| *ACE_ID* | NA | NA | 0.679 | 0.203 | 0.099 | 0.524 | 0.537 | 0.514 |
| *AGT M235T* | NA | NA | NA | NA | 0.881 | 0.987 | 0.579 | NA |
| *PAI- 1 4G5G* | 0.679 | NA | NA | NA | 0.957 | 0.526 | 0.706 | 0.317 |
| *REN G/T rs1464816* | 0.203 | NA | NA | NA | 0.422 | 0.874 | 0.300 | 0.932 |
| ETNK2 A/G  *rs1917542* | 0.099 | 0.881 | 0.957 | 0.422 | NA | NA | 0.308 | 0.966 |
| ETNK2 C/T  *rs2293337* | 0.524 | 0.987 | 0.526 | 0.874 | NA | NA | 0.813 | 0.877 |
| REN T/C  *rs3730103* | 0.537 | 0.579 | 0.706 | 0.300 | 0.308 | 0.813 | NA | 0.143 |
| *t-PA ID* | 0.514 | NA | 0.317 | 0.932 | 0.966 | 0.877 | 0.143 | NA |
|  |  |  |  |  |  |  |  |  |
| **RxA** | *ACE ID* | AGT  *M235T* | *PAI-1 4G5G* | REN G/T  *rs1464816* | ETNK2 A/G  *rs1917542* | ETNK2 C/T  *rs2293337* | REN T/C  *rs3730103* | *t-PA ID* |
| *ACE_ID* | NA | 0.516 | 0.216 | 0.314 | 0.754 | 0.183 | 0.921 | 0.118 |
| *AGT M235T* | 0.420 | NA | 0.507 | 0.167 | **0.086** | 0.880 | 0.750 | 0.733 |
| *PAI- 1 4G5G* | 0.282 | 0.487 | NA | 0.159 | **0.028** | 0.320 | 0.665 | 0.359 |
| *REN G/T rs1464816* | 0.250 | **0.027** | 0.467 | NA | 0.155 | 0.654 | 0.804 | 0.186 |
| ETNK2 A/G  *rs1917542* | **0.095** | 0.183 | 0.103 | 0.290 | NA | 0.147 | 0.423 | 0.754 |
| ETNK2 C/T  *rs2293337* | 0.403 | 0.835 | 0.697 | 0.543 | 0.333 | NA | 0.173 | 0.941 |
| REN T/C  *rs3730103* | 0.569 | 0.559 | 0.728 | 0.707 | 0.396 | 0.189 | NA | 0.980 |
| *t-PA ID* | **0.031** | 0.791 | **0.045** | 0.152 | 0.218 | 0.988 | **0.016** | NA |
|  |  |  |  |  |  |  |  |  |
| **RxR** | *ACE ID* | AGT  *M235T* | *PAI-1 4G5G* | REN G/T  *rs1464816* | ETNK2 A/G  *rs1917542* | ETNK2 C/T  *rs2293337* | REN T/C  *rs3730103* | *t-PA ID* |
| *ACE_ID* | NA | 0.342 | 0.200 | 0.332 | 0.457 | 0.201 | 0.830 | **0.042** |
| *AGT M235T* | 0.342 | NA | 0.264 | 0.237 | **0.067** | 0.587 | 0.713 | 0.499 |
| *PAI- 1 4G5G* | 0.200 | 0.264 | NA | 0.238 | **0.038** | 0.822 | 0.438 | 0.365 |
| *REN G/T rs1464816* | 0.332 | 0.237 | 0.238 | NA | 0.551 | 0.771 | 0.537 | **0.073** |
| ETNK2 A/G  *rs1917542* | 0.457 | 0.067 | 0.038 | 0.551 | NA | 0.334 | 0.206 | 0.920 |
| ETNK2 C/T  *rs2293337* | 0.201 | 0.587 | 0.822 | 0.771 | 0.334 | NA | 0.206 | 0.920 |
| REN T/C  *rs3730103* | 0.830 | 0.713 | 0.438 | 0.537 | 0.206 | 0.110 | NA | 0.933 |
| *t-PA ID* | 0.042 | 0.499 | 0.365 | 0.073 | 0.920 | 0.971 | 0.933 | NA |

|  |  |  |  |  |  |  |  |  |
| --- | --- | --- | --- | --- | --- | --- | --- | --- |
| **RxD** | *ACE ID* | AGT  *M235T* | *PAI-1 4G5G* | REN G/T  *rs1464816* | ETNK2 A/G  *rs1917542* | ETNK2 C/T  *rs2293337* | REN T/C  *rs3730103* | *t-PA ID* |
| *ACE_ID* | NA | 0.350 | 0.483 | 0.146 | 0.854 | **0.090** | 0.691 | 0.342 |
| *AGT M235T* | 0.484 | NA | 0.665 | 0.235 | NA | 0.730 | 0.527 | 0.617 |
| *PAI- 1 4G5G* | 0.195 | 0.623 | NA | 0.327 | **0.030** | 0.138 | 0.822 | 0.465 |
| *REN G/T rs1464816* | 0.299 | **0.008** | 0.443 | NA | **0.067** | 0.384 | 0.680 | 0.343 |
| ETNK2 A/G  *rs1917542* | **0.032** | 0.615 | 0.254 | 0.247 | NA | **0.081** | 0.583 | 0.472 |
| ETNK2 C/T  *rs2293337* | 0.994 | 0.711 | 0.483 | 0.357 | NA | NA | 0.632 | 0.731 |
| REN T/C  *rs3730103* | 0.299 | 0.430 | 0.723 | 0.482 | 0.889 | 0.524 | NA | 0.921 |
| *t-PA ID* | **0.037** | 0.915 | **0.052** | 0.235 | 0.103 | 0.934 | **0.005** | NA |
|  |  |  |  |  |  |  |  |  |
| **AxA** | *ACE ID* | AGT  *M235T* | *PAI-1 4G5G* | REN G/T  *rs1464816* | ETNK2 A/G  *rs1917542* | ETNK2 C/T  *rs2293337* | REN T/C  *rs3730103* | *t-PA ID* |
| *ACE_ID* | NA | 0.520 | 0.394 | 0.190 | 0.213 | 0.341 | 0.586 | 0.132 |
| *AGT M235T* | 0.520 | NA | 0.666 | **0.035** | 0.389 | 0.954 | 0.500 | 0.899 |
| *PAI- 1 4G5G* | 0.394 | 0.666 | NA | 0.281 | **0.091** | 0.430 | 0.905 | **0.025** |
| *REN G/T rs1464816* | 0.190 | 0.035 | 0.281 | NA | 0.172 | 0.742 | 0.539 | 0.364 |
| ETNK2 A/G  *rs1917542* | 0.213 | 0.389 | 0.091 | 0.172 | NA | 0.158 | 0.584 | 0.436 |
| ETNK2 C/T  *rs2293337* | 0.341 | 0.954 | 0.430 | 0.742 | 0.158 | NA | 0.285 | 0.996 |
| REN T/C  *rs3730103* | 0.586 | 0.500 | 0.905 | 0.539 | 0.584 | 0.285 | NA | **0.072** |
| *t-PA ID* | 0.132 | 0.899 | 0.025 | 0.364 | 0.436 | 0.996 | 0.072 | NA |
|  |  |  |  |  |  |  |  |  |
| **AxD** | *ACE ID* | AGT  *M235T* | *PAI-1 4G5G* | REN G/T  *rs1464816* | ETNK2 A/G  *rs1917542* | ETNK2 C/T  *rs2293337* | REN T/C  *rs3730103* | *t-PA ID* |
| *ACE_ID* | NA | 0.297 | 0.640 | 0.236 | 0.242 | 0.137 | 0.670 | 0.587 |
| *AGT M235T* | 0.467 | NA | 0.654 | 0.240 | 0.899 | 0.905 | 0.568 | 0.655 |
| *PAI- 1 4G5G* | 0.441 | 0.603 | NA | 0.312 | **0.082** | 0.168 | 0.928 | 0.342 |
| *REN G/T rs1464816* | 0.131 | **0.010** | 0.408 | NA | 0.155 | 0.684 | 0.415 | 0.659 |
| ETNK2 A/G  *rs1917542* | **0.061** | 0.869 | 0.479 | 0.237 | NA | **0.092** | 0.613 | 0.788 |
| ETNK2 C/T  *rs2293337* | 0.813 | 0.984 | 0.717 | 0.591 | NA | NA | 0.717 | 0.933 |
| REN T/C  *rs3730103* | 0.327 | 0.436 | 0.808 | 0.305 | 0.609 | 0.751 | NA | 0.287 |
| *t-PA ID* | 0.113 | 0.867 | **0.055** | 0.411 | 0.215 | 0.962 | **0.018** | NA |
|  |  |  |  |  |  |  |  |  |
| **AxR** | *ACE ID* | AGT  *M235T* | *PAI-1 4G5G* | REN G/T  *rs1464816* | ETNK2 A/G  *rs1917542* | ETNK2 C/T  *rs2293337* | REN T/C  *rs3730103* | *t-PA ID* |
| *ACE_ID* | NA | 0.420 | 0.282 | 0.250 | **0.095** | 0.403 | 0.569 | **0.031** |
| *AGT M235T* | 0.516 | NA | 0.487 | **0.027** | 0.183 | 0.835 | 0.559 | 0.791 |
| *PAI- 1 4G5G* | 0.216 | 0.507 | NA | 0.467 | 0.103 | 0.697 | 0.728 | **0.045** |
| *REN G/T rs1464816* | 0.314 | 0.167 | 0.159 | NA | 0.290 | 0.543 | 0.707 | 0.152 |
| ETNK2 A/G  *rs1917542* | 0.754 | **0.086** | **0.028** | 0.155 | NA | 0.333 | 0.396 | 0.218 |
| ETNK2 C/T  *rs2293337* | 0.183 | 0.880 | 0.320 | 0.654 | 0.147 | NA | 0.189 | 0.988 |
| REN T/C  *rs3730103* | 0.921 | 0.750 | 0.665 | 0.804 | 0.423 | 0.173 | NA | **0.016** |
| *t-PA ID* | 0.118 | 0.733 | 0.359 | 0.186 | 0.754 | 0.941 | 0.980 | NA |
