# Supplementary material for: Epistatic Interactions in Genetic Regulation of t-PA and PAI-1 Levels in a Ghanaian Population
Source: PLoS One. 2011 Jan 31;6(1):e16639. doi: 10.1371/journal.pone.0016639 (PMC3031598; doi:10.1371/journal.pone.0016639)
Supplement: Table S5 — p -values for epistatic effects between polymorphisms in association with plasma PAI-1 levels for males. All nine interaction models are presented. Each table displays the results of one interaction pairing as indicated in the upper left-hand corner. The notation is column by row, for example, a DxA interaction indicates that the SNPs across the columns are encoded as dominant while the SNPs down the rows are endoded as additive. p-values <0.10 are displayed in boldface. D = dominant, A = additive, R = recessive. (DOC) [file pone.0016639.s005.doc]

**Table S5.**

| **DxA** | *ACE ID* | AGT  *M235T* | *PAI-1 4G5G* | REN G/T  *rs1464816* | ETNK2 A/G  *rs1917542* | ETNK2 C/T  *rs2293337* | REN T/C  *rs3730103* | *t-PA ID* |
| --- | --- | --- | --- | --- | --- | --- | --- | --- |
| *ACE_ID* | NA | 0.800 | 0.539 | 0.369 | 0.144 | 0.927 | 0.387 | 0.373 |
| *AGT M235T* | 0.887 | NA | 0.907 | 0.313 | 0.787 | 0.994 | 0.528 | 0.928 |
| *PAI- 1 4G5G* | 0.355 | 0.849 | NA | 0.527 | 0.774 | 0.734 | 0.526 | 0.473 |
| REN G/T  *rs1464816* | 0.347 | 0.764 | 0.916 | NA | 0.528 | 0.600 | 0.727 | 0.846 |
| ETNK2 A/G  *rs1917542* | 0.478 | 0.909 | 0.238 | 0.367 | NA | NA | 0.948 | 0.434 |
| ETNK2 C/T  *rs2293337* | 0.426 | 0.992 | 0.429 | 0.435 | 0.408 | NA | 0.848 | 0.885 |
| REN T/C  *rs3730103* | 0.655 | 0.991 | 0.547 | 0.604 | 0.909 | 0.928 | NA | 0.992 |
| *t-PA ID* | 0.302 | 0.995 | 0.457 | 0.942 | 0.878 | 0.815 | 0.822 | NA |
|  |  |  |  |  |  |  |  |  |
| **DxR** | *ACE ID* | AGT  *M235T* | *PAI-1 4G5G* | REN G/T  *rs1464816* | ETNK2 A/G  *rs1917542* | ETNK2 C/T  *rs2293337* | REN T/C  *rs3730103* | *t-PA ID* |
| *ACE_ID* | NA | 0.824 | 0.278 | 0.827 | **0.058** | 0.817 | 0.496 | 0.944 |
| *AGT M235T* | 0.882 | NA | 0.911 | 0.311 | 0.494 | 0.978 | 0.292 | 0.939 |
| *PAI- 1 4G5G* | 0.144 | 0.822 | NA | 0.440 | 0.551 | 0.610 | 0.381 | 0.670 |
| REN G/T  *rs1464816* | 0.703 | 0.756 | 0.928 | NA | 0.577 | 0.358 | 0.516 | 0.540 |
| ETNK2 A/G  *rs1917542* | 0.993 | NA | **0.091** | 0.220 | NA | NA | 0.845 | 0.189 |
| ETNK2 C/T  *rs2293337* | 0.251 | 0.939 | 0.210 | 0.374 | 0.334 | NA | 0.640 | 0.783 |
| REN T/C  *rs3730103* | 0.539 | 0.883 | 0.415 | 0.408 | 0.688 | 0.550 | NA | 0.869 |
| *t-PA ID* | 0.244 | 0.959 | 0.292 | 0.934 | 0.770 | 0.491 | 0.501 | NA |

|  |  |  |  |  |  |  |  |  |
| --- | --- | --- | --- | --- | --- | --- | --- | --- |
| **DxD** | *ACE ID* | AGT  *M235T* | *PAI-1 4G5G* | REN G/T  *rs1464816* | ETNK2 A/G  *rs1917542* | ETNK2 C/T  *rs2293337* | REN T/C  *rs3730103* | *t-PA ID* |
| *ACE_ID* | NA | NA | 0.831 | 0.165 | 0.244 | 0.764 | 0.388 | 0.198 |
| *AGT M235T* | NA | NA | NA | NA | 0.885 | 0.994 | 0.992 | NA |
| *PAI- 1 4G5G* | 0.831 | NA | NA | NA | 0.591 | 0.464 | 0.350 | 0.328 |
| REN G/T  *rs1464816* | 0.165 | NA | NA | NA | 0.469 | 0.520 | 0.819 | 0.752 |
| ETNK2 A/G  *rs1917542* | 0.244 | 0.885 | 0.591 | 0.469 | NA | NA | 0.788 | 0.795 |
| ETNK2 C/T  *rs2293337* | 0.764 | 0.994 | 0.464 | 0.520 | NA | NA | 0.849 | 0.858 |
| REN T/C  *rs3730103* | 0.388 | 0.992 | 0.350 | 0.819 | 0.788 | 0.849 | NA | 0.971 |
| *t-PA ID* | 0.198 | NA | 0.328 | 0.752 | 0.795 | 0.858 | 0.971 | NA |
|  |  |  |  |  |  |  |  |  |
| **RxA** | *ACE ID* | AGT  *M235T* | *PAI-1 4G5G* | REN G/T  *rs1464816* | ETNK2 A/G  *rs1917542* | ETNK2 C/T  *rs2293337* | REN T/C  *rs3730103* | *t-PA ID* |
| *ACE_ID* | NA | 0.818 | 0.341 | 0.783 | 0.984 | 0.404 | 0.328 | 0.440 |
| *AGT M235T* | 0.818 | NA | 0.967 | 0.827 | 0.285 | 0.973 | 0.983 | 0.957 |
| *PAI- 1 4G5G* | 0.537 | 0.980 | NA | 0.874 | 0.162 | 0.324 | 0.695 | 0.537 |
| REN G/T  *rs1464816* | 0.766 | 0.579 | 0.568 | NA | 0.127 | 0.380 | 0.451 | **0.082** |
| ETNK2 A/G  *rs1917542* | 0.110 | 0.320 | 0.459 | **0.062** | NA | 0.705 | 0.877 | 0.869 |
| ETNK2 C/T  *rs2293337* | 0.544 | 0.974 | 0.745 | 0.168 | 0.755 | NA | 0.687 | 0.774 |
| REN T/C  *rs3730103* | 0.140 | 0.553 | 0.679 | 0.636 | 0.913 | 0.571 | NA | 0.716 |
| *t-PA ID* | 0.600 | 0.966 | 0.868 | **0.098** | 0.412 | 0.802 | 0.869 | NA |

|  |  |  |  |  |  |  |  |  |
| --- | --- | --- | --- | --- | --- | --- | --- | --- |
| **RxR** | *ACE ID* | AGT  *M235T* | *PAI-1 4G5G* | REN G/T  *rs1464816* | ETNK2 A/G  *rs1917542* | ETNK2 C/T  *rs2293337* | REN T/C  *rs3730103* | *t-PA ID* |
| *ACE_ID* | NA | 0.595 | 0.734 | 0.472 | 0.868 | 0.276 | 0.135 | 0.354 |
| *AGT M235T* | 0.595 | NA | 0.942 | 0.661 | 0.292 | 0.840 | 0.946 | 0.792 |
| *PAI- 1 4G5G* | 0.734 | 0.942 | NA | 0.706 | 0.218 | 0.704 | 0.658 | 0.927 |
| REN G/T  *rs1464816* | 0.472 | 0.661 | 0.706 | NA | **0.062** | 0.203 | 0.542 | **0.032** |
| ETNK2 A/G  *rs1917542* | 0.868 | 0.292 | 0.218 | 0.062 | NA | 0.765 | 0.669 | 0.738 |
| ETNK2 C/T  *rs2293337* | 0.276 | 0.840 | 0.704 | 0.203 | 0.765 | NA | 0.434 | 0.682 |
| REN T/C  *rs3730103* | 0.135 | 0.946 | 0.658 | 0.542 | 0.669 | 0.434 | NA | 0.566 |
| *t-PA ID* | 0.354 | 0.792 | 0.927 | 0.032 | 0.738 | 0.682 | 0.566 | NA |
|  |  |  |  |  |  |  |  |  |
| **RxD** | *ACE ID* | AGT  *M235T* | *PAI-1 4G5G* | REN G/T  *rs1464816* | ETNK2 A/G  *rs1917542* | ETNK2 C/T  *rs2293337* | REN T/C  *rs3730103* | *t-PA ID* |
| *ACE_ID* | NA | 0.882 | 0.144 | 0.703 | 0.993 | 0.251 | 0.539 | 0.244 |
| *AGT M235T* | 0.824 | NA | 0.822 | 0.756 | NA | 0.939 | 0.883 | 0.959 |
| *PAI- 1 4G5G* | 0.278 | 0.911 | NA | 0.928 | **0.091** | 0.210 | 0.415 | 0.292 |
| REN G/T  *rs1464816* | 0.827 | 0.311 | 0.440 | NA | 0.220 | 0.374 | 0.408 | 0.934 |
| ETNK2 A/G  *rs1917542* | **0.058** | 0.494 | 0.551 | 0.577 | NA | 0.334 | 0.688 | 0.770 |
| ETNK2 C/T  *rs2293337* | 0.817 | 0.978 | 0.610 | 0.358 | NA | NA | 0.550 | 0.491 |
| REN T/C  *rs3730103* | 0.496 | 0.292 | 0.381 | 0.516 | 0.845 | 0.640 | NA | 0.501 |
| *t-PA ID* | 0.944 | 0.939 | 0.670 | 0.540 | 0.189 | 0.783 | 0.869 | NA |

|  |  |  |  |  |  |  |  |  |
| --- | --- | --- | --- | --- | --- | --- | --- | --- |
| **AxA** | *ACE ID* | AGT  *M235T* | *PAI-1 4G5G* | REN G/T  *rs1464816* | ETNK2 A/G  *rs1917542* | ETNK2 C/T  *rs2293337* | REN T/C  *rs3730103* | *t-PA ID* |
| *ACE_ID* | NA | 0.904 | 0.494 | 0.616 | 0.322 | 0.700 | 0.243 | 0.513 |
| *AGT M235T* | 0.904 | NA | 0.992 | 0.750 | 0.503 | 0.994 | 0.852 | 0.994 |
| *PAI- 1 4G5G* | 0.494 | 0.992 | NA | 0.778 | 0.451 | 0.608 | 0.806 | 0.710 |
| REN G/T  *rs1464816* | 0.616 | 0.750 | 0.778 | NA | 0.147 | 0.382 | 0.612 | 0.290 |
| ETNK2 A/G  *rs1917542* | 0.322 | 0.503 | 0.451 | 0.147 | NA | 0.769 | 0.988 | 0.746 |
| ETNK2 C/T  *rs2293337* | 0.700 | 0.994 | 0.608 | 0.382 | 0.769 | NA | 0.872 | 0.936 |
| REN T/C  *rs3730103* | 0.243 | 0.852 | 0.806 | 0.612 | 0.988 | 0.872 | NA | 0.964 |
| *t-PA ID* | 0.513 | 0.994 | 0.710 | 0.290 | 0.746 | 0.936 | 0.964 | NA |
|  |  |  |  |  |  |  |  |  |
| **AxD** | *ACE ID* | AGT  *M235T* | *PAI-1 4G5G* | REN G/T  *rs1464816* | ETNK2 A/G  *rs1917542* | ETNK2 C/T  *rs2293337* | REN T/C  *rs3730103* | *t-PA ID* |
| *ACE_ID* | NA | 0.887 | 0.355 | 0.347 | 0.478 | 0.426 | 0.655 | 0.302 |
| *AGT M235T* | 0.800 | NA | 0.849 | 0.764 | 0.909 | 0.992 | 0.991 | 0.995 |
| *PAI- 1 4G5G* | 0.539 | 0.907 | NA | 0.916 | 0.238 | 0.429 | 0.547 | 0.457 |
| REN G/T  *rs1464816* | 0.369 | 0.313 | 0.527 | NA | 0.367 | 0.435 | 0.604 | 0.942 |
| ETNK2 A/G  *rs1917542* | 0.144 | 0.787 | 0.774 | 0.528 | NA | 0.408 | 0.909 | 0.878 |
| ETNK2 C/T  *rs2293337* | 0.927 | 0.994 | 0.734 | 0.600 | NA | NA | 0.928 | 0.815 |
| REN T/C  *rs3730103* | 0.387 | 0.528 | 0.526 | 0.727 | 0.948 | 0.848 | NA | 0.822 |
| *t-PA ID* | 0.373 | 0.928 | 0.473 | 0.846 | 0.434 | 0.885 | 0.992 | NA |

|  |  |  |  |  |  |  |  |  |
| --- | --- | --- | --- | --- | --- | --- | --- | --- |
| **AxR** | *ACE ID* | AGT  *M235T* | *PAI-1 4G5G* | REN G/T  *rs1464816* | ETNK2 A/G  *rs1917542* | ETNK2 C/T  *rs2293337* | REN T/C  *rs3730103* | *t-PA ID* |
| *ACE_ID* | NA | 0.818 | 0.537 | 0.766 | 0.110 | 0.544 | 0.140 | 0.600 |
| *AGT M235T* | 0.818 | NA | 0.980 | 0.579 | 0.320 | 0.974 | 0.553 | 0.966 |
| *PAI- 1 4G5G* | 0.341 | 0.967 | NA | 0.568 | 0.459 | 0.745 | 0.679 | 0.868 |
| REN G/T  *rs1464816* | 0.783 | 0.827 | 0.874 | NA | **0.062** | 0.168 | 0.636 | **0.098** |
| ETNK2 A/G  *rs1917542* | 0.984 | 0.285 | 0.162 | 0.127 | NA | 0.755 | 0.913 | 0.412 |
| ETNK2 C/T  *rs2293337* | 0.404 | 0.973 | 0.324 | 0.380 | 0.705 | NA | 0.571 | 0.802 |
| REN T/C  *rs3730103* | 0.328 | 0.983 | 0.695 | 0.451 | 0.877 | 0.687 | NA | 0.869 |
| *t-PA ID* | 0.440 | 0.957 | 0.537 | **0.082** | 0.869 | 0.774 | 0.716 | NA |
