# Supplementary material for: Epistatic Interactions in Genetic Regulation of t-PA and PAI-1 Levels in a Ghanaian Population
Source: PLoS One. 2011 Jan 31;6(1):e16639. doi: 10.1371/journal.pone.0016639 (PMC3031598; doi:10.1371/journal.pone.0016639)
Supplement: Table S6 — Description of the data underlying the points in the interaction plots displayed in Figure 5 . The mean protein level, 95% confidence interval and number of individuals for each genotype combination (i.e. each data point of interaction plot) is presented. (DOC) [file pone.0016639.s006.doc]

**Supplemental Table 6.**

| ***Female ln t-PA*** | **ETNK2:rs1917542 (A6135G)** | | | | | | | | | | | |
| --- | --- | --- | --- | --- | --- | --- | --- | --- | --- | --- | --- | --- |
|  |  | AA (0) | | |  | AG (1) | | |  | GG (2) | | |
|  |  | Mean | ± (95% CI) | N^a^ |  | Mean | ± (95% CI) | N |  | Mean | ± (95% CI) | N |
| **REN:rs3730103**  **(T9435C)** | CC (2) | 1.88 | 0.26 | 10 |  | 1.44 | 0.39 | 25 |  | 1.95 | 0.21 | 19 |
|  | TC (1) | 1.90 | 0.14 | 90 |  | 1.84 | 0.12 | 129 |  | 1.70 | 0.23 | 42 |
|  | TT (0) | 1.70 | 0.12 | 106 |  | 1.85 | 0.12 | 121 |  | 1.61 | 0.26 | 28 |
|  | | | | | | | | | | | | |
| ***Femaleln PAI-1*** | **REN:rs3730103 (T9435C)** | | | | | | | | | | | |
|  |  | CC (2) | | |  | TC (1) | | |  | TT (0) | | |
|  |  | Mean | ± (95% CI) | N |  | Mean | ± (95% CI) | N |  | Mean | ± (95% CI) | N |
| **TPA:rs4646972** | DD (0) | 0.73 | 0.79 | 24 |  | 1.21 | 0.24 | 115 |  | 1.34 | 0.23 | 102 |
|  | DI (1) | 0.67 | 0.50 | 18 |  | 1.44 | 0.21 | 120 |  | 1.18 | 0.26 | 116 |
|  | II (2) | 2.25 | 0.68 | 12 |  | 1.23 | 0.53 | 26 |  | 1.28 | 0.54 | 37 |
|  | | | | | | | | | | | | |
| ***Male ln t-PA*** | **REN:rs3730103 (T9435C)** | | | | | | | | | | | |
|  |  | CC (2) | | |  | TC (1) | | |  | TT (0) | | |
|  |  | Mean | ± (95% CI) | N |  | Mean | ± (95% CI) | N |  | Mean | ± (95% CI) | N |
| **TPA:rs4646972** | DD (0) | 1.99 | 0.24 | 23 |  | 1.90 | 0.16 | 72 |  | 1.77 | 0.21 | 68 |
|  | DI (1) | 1.80 | 0.25 | 21 |  | 1.81 | 0.14 | 89 |  | 1.76 | 0.15 | 106 |
|  | II (2) | 2.07 | 0.28 | 6 |  | 1.81 | 0.31 | 18 |  | 1.07 | 0.76 | 19 |
|  | | | | | | | | | | | | |
| **Male ln PAI-1** | **REN:rs1464819 (G6567T)** | | | | | | | | | | | |
|  | GG (0) | | | |  | GT (1) | | |  | TT (2) | | |
|  |  | Mean | ± (95% CI) | N |  | Mean | ± (95% CI) | N |  | Mean | ± (95% CI) | N |
|  | DD (0) | 1.00 | 0.34 | 71 |  | 1.13 | 0.33 | 78 |  | 0.83 | 1.08 | 14 |
| **TPA:rs4646972** | ID (1) | 1.30 | 0.29 | 82 |  | 0.72 | 0.30 | 102 |  | 0.78 | 0.59 | 32 |
|  | II (2) | 1.83 | 0.64 | 16 |  | 1.30 | 0.90 | 17 |  | 1.15 | 0.50 | 10 |
